# Light-induced Variation in Phenolic Compounds in Cabernet Sauvignon Grapes (*Vitis vinifera* L.) Involves Extensive Transcriptome Reprogramming of Biosynthetic Enzymes, Transcription Factors, and Phytohormonal Regulators

**DOI:** 10.3389/fpls.2017.00547

**Published:** 2017-04-19

**Authors:** Run-Ze Sun, Guo Cheng, Qiang Li, Yan-Nan He, Yu Wang, Yi-Bin Lan, Si-Yu Li, Yan-Rong Zhu, Wen-Feng Song, Xue Zhang, Xiao-Di Cui, Wu Chen, Jun Wang

**Affiliations:** ^1^Center for Viticulture and Enology, College of Food Science and Nutritional Engineering, China Agricultural UniversityBeijing, China; ^2^Key Laboratory of Viticulture and Enology, Ministry of AgricultureBeijing, China; ^3^College of Enology, Northwest A&F UniversityYangling, China; ^4^CITIC Guoan Wine Co. Ltd.Xinjiang, China; ^5^Key Laboratory of Plant Resources and Beijing Botanical Garden, Institute of Botany, Chinese Academy of ScienceBeijing, China; ^6^Grape and Wine Research Institute, Guangxi Academy of Agricultural SciencesNanning, China; ^7^Institute of Vegetables and Flowers, Chinese Academy of Agricultural SciencesBeijing, China

**Keywords:** *Vitis vinifera*, sunlight exposure, phenolic compounds, transcriptome, transcription factor, phytohormone signaling

## Abstract

Light environments have long been known to influence grape (*Vitis vinifera* L.) berry development and biosynthesis of phenolic compounds, and ultimately affect wine quality. Here, the accumulation and compositional changes of hydroxycinnamic acids (HCAs) and flavonoids, as well as global gene expression were analyzed in Cabernet Sauvignon grape berries under sunlight exposure treatments at different phenological stages. Sunlight exposure did not consistently affect the accumulation of berry skin flavan-3-ol or anthocyanin among different seasons due to climatic variations, but increased HCA content significantly at véraison and harvest, and enhanced flavonol accumulation dramatically with its timing and severity degree trend. As in sunlight exposed berries, a highly significant correlation was observed between the expression of genes coding phenylalanine ammonia-lyase, 4-coumarate: CoA ligase, flavanone 3-hydroxylase and flavonol synthase family members and corresponding metabolite accumulation in the phenolic biosynthesis pathway, which may positively or negatively be regulated by MYB, bHLH, WRKY, AP2/EREBP, C2C2, NAC, and C2H2 transcription factors (TFs). Furthermore, some candidate genes required for auxin, ethylene and abscisic acid signal transductions were also identified which are probably involved in berry development and flavonoid biosynthesis in response to enhanced sunlight irradiation. Taken together, this study provides a valuable overview of the light-induced phenolic metabolism and transcriptome changes, especially the dynamic responses of TFs and signaling components of phytohormones, and contributes to the further understanding of sunlight-responsive phenolic biosynthesis regulation in grape berries.

## Introduction

Phenolic compounds, mainly hydroxycinnamic acids (HCAs) and flavonoids, are one of the most abundant secondary metabolites in grape (*Vitis vinifera* L.) berries and important to wine quality. HCAs accumulated in grape berry skin and flesh are *p*-coumaric, caffeic, ferulic, sinapic acid and their derivatives, usually in the form of esters (Baderschneider and Winterhalter, 2001). Three major classes of flavonoid compounds found in grapes include proanthocyanidins (PAs), anthocyanins and flavonols. PAs, also named condensed tannins, are polymers of flavan-3-ol monomeric units (such as catechin, epicatechin, epicatechin-3-*O*-gallte, and epigallocatechin) which located in both the grape skins and the seeds, with trace amounts also accumulated in the vasculature of berries, whereas flavonols and anthocyanins are detected only in berry skins (Downey et al., 2006). All these compounds have important physiological functions in diverse aspects of grape berry development, such as free radical scavenging, pigmentation and co-pigmentation, ultraviolet (UV) radiation protection and defense against microbial and fungal infections (Harborne and Williams, 2000; Winkel-Shirley, 2001). Furthermore, their contribution to the color, bitterness, astringency, and antioxidant properties of red wine and potential benefits for human health have gained much attention on elucidating the regulatory mechanism of phenolic biosynthesis in grapes over the years (Santos-Buelga and Scalbert, 2000; Conde et al., 2007). Phenolic compounds are derived from multiple branches of the phenylpropanoid biosynthetic pathway, one of the secondary metabolic routes well-characterized in diverse plant species (Hahlbrock and Scheel, 1989; Ferrer et al., 2008). The genes encoding the enzymes of the phenolic biosynthesis pathway in grapes have been isolated (Sparvoli et al., 1994) and also predicted from the complete genome sequence (Velasco et al., 2007; Da Silva et al., 2013), nearly all of which are composed of small gene families. The transcriptional regulation of some structural genes is mainly controlled by a ternary complex (MBW) involving transcription factors (TFs) from the R2R3-MYB, MYC-like basic helix-loop-helix (bHLH), and tryptophan-aspartic acid repeat (WDR, also known as WD40) proteins in several plant species, including *V*. *vinifera* (Hichri et al., 2011). Additional potential regulators of the phenolic biosynthesis pathway have also been identified in model and crop plants, such as the *Arabidopsis* WRKY, MADS (MCM1, Agamous, Deficiens, serum response factor) box and bZIP (basic domain/leucine zipper) TFs, as well as the maize R-Interacting Factor 1 (RIF1), an EMSY-related protein interacted with a certain bHLH protein, ZmR (Hichri et al., 2011). Furthermore, several other R2R3-MYB and single-repeat R3-MYB proteins, such as *Arabidopsis* AtMYBL2 and CPC (CAPRICE), gentian GtMYB1R1 and GtMYB1R9 and strawberry FaMYB1, act as transcriptional repressors which negatively regulate the biosynthesis of anthocyanins or PAs in plants (Aharoni et al., 2001; Matsui et al., 2008; Zhu et al., 2009; Nakatsuka et al., 2013). In the case of grapevine, VviMYB4a and its close homolog VviMYB4b have been characterized as important negative regulators of small-weight phenolic biosynthesis, whereas two other repressors, VviMYBC2-L1 and VviMYBC2-L3, were shown to fine tune flavonoid levels additionally (Huang et al., 2014; Cavallini et al., 2015).

Environmental factors (light, temperature, water status, and nutrients, etc.) and viticulture practices have been acknowledged to influence the development, ripening and phenolics composition of grape berries, and could thereby affect wine quality (Jackson and Lombard, 1993; Downey et al., 2006). Bunch shading and exposure treatments are regarded as influential practices that alter the accumulation and composition of phenolics and the expression of the corresponding biosynthetic genes by directly affecting the incidence of light on grape clusters and also changing other microclimatic aspects, such as temperature and humidity (Ristic et al., 2007; Koyama and Goto-Yamamoto, 2008; Chorti et al., 2010). Many studies have shown that artificial bunch shading resulted in greatly decreased flavonol concentrations, while the levels of PAs and anthocyanins were not significantly changed at harvest (Downey et al., 2004; Fujita et al., 2005; Cortell and Kennedy, 2006; Koyama and Goto-Yamamoto, 2008). On the other hand, enhanced sunlight exposure induced by basal leaf removal generally led to increased accumulation of flavonols, but did not alter anthocyanin concentration compared with the control, which might be correlated with the negative effects of elevated berry skin temperature (Downey et al., 2004; Chorti et al., 2010). In addition, grapes from sunlight exposure bunches had a higher proportion of B-ring trihydroxylation subunits within PAs and anthocyanins in comparison with normal and bunch shading fruit, which agree with the relative increase of *flavonoid 3′,5′-hydroxylase* (*VviF3′5′H*) expression (Cortell and Kennedy, 2006; Koyama and Goto-Yamamoto, 2008).

In recent years, a considerable amount of effort has been devoted to investigating the impact of cluster sunlight exposure treatments during specific stages of berry development and ripening on the detailed phenolic profiles as well as the expression of related structural and regulatory genes in different grape varieties (Matus et al., 2009; Chorti et al., 2010; Lemut et al., 2011; Kotseridis et al., 2012; Lee and Skinkis, 2013; Matsuyama et al., 2014; Wu et al., 2014; Friedel et al., 2015). For instance, the expression of *flavonol synthase 1* (*VviFLS1*, also known as *VviFLS4*) and its specific transcriptional activator *VviMYB12* (also named *VviMYBF1*) was drastically increased following leaf removal treatment, which ultimately resulted in the quickly increased flavonol synthesis (Matus et al., 2009). Leaf removal also up-regulated anthocyanin synthesis related structural genes and regulators in grape skins, such as *chalcone synthase* (*VviCHS*), *uridine diphosphate (UDP)-glucose:flavonoid 3-O-glucosyltransferase* (*VviUFGT*), *anthocyanin-O-methyltransferase* (*VviAOMT*), *flavonoid 3′-hydroxylase* (*VviF3′H*), *VviF3′5′H, VviMYBA1*, and *VviMYB5a* (Matus et al., 2009; Matsuyama et al., 2014; Wu et al., 2014). More recently, two bZIP TFs elongated hypocotyl 5 protein (HY5) orthologs, VviHY5 and VviHYH, were characterized as constituents of the UV-B response pathway in grapevine and mediated flavonol accumulation in response to high radiation exposure (Loyola et al., 2016; Matus, 2016). However, there are still pending questions regarding the complex underlying molecular mechanism of the phenolic metabolism regulation network involved in light response. In the present study, accumulation and compositional changes of HCAs, flavan-3-ols, anthocyanins and flavonols were determined in *V. vinifera* L. cv. Cabernet Sauvignon grape berries from different fruit-zone light-exposure treatments in multiple phenological stages under field conditions over three successive seasons. To understand the regulation of phenolic biosynthesis under different irradiation conditions, the influences of light exposure on the transcription of phenolic biosynthetic genes and their putative upstream regulators, as well as the relationship between metabolism and transcription in grapes throughout berry development were also examined.

## Materials and Methods

### Plant Material and Sunlight Exposure Treatment

Field experiments were conducted in a commercial vineyard of *V. vinifera* L. Cabernet Sauvignon located in Manas Country (44°17′ North, 86°12*′* East, 475 m above sea level), the wine-producing region of Xinjiang province, China, for three consecutive growing seasons (2011, 2012, and 2013). The own-rooted vines in this vineyard were planted in 2000, managed on a modified Vertical-Shoot-Positioned (M-VSP) trellis system with a spur-pruned cordon retaining 15 nodes per linear meter, arranged in north-south rows with 2.5 m × 1 m vine spacing and equipped with a furrow irrigated system. Nutrition and pest management was carried out according to industry standards for this cultivar and the region as previously described (Cheng et al., 2014).

**FIGURE 1:**
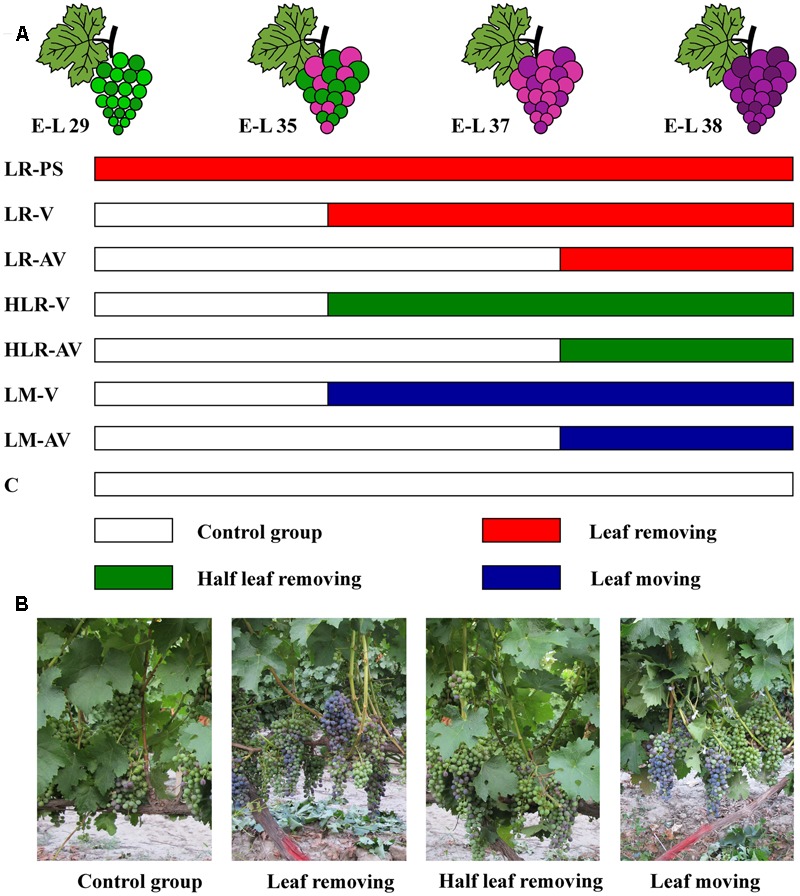
**(A)** Experimental design for different cluster sunlight exposure treatments and **(B)** field photographs of Cabernet Sauvignon grapes under different sunlight exposure treatments. LR-PS, leaf removal at berry pepper-corn size; LR-V, leaf removal at véraison; LR-AV, leaf removal after véraison; HLR-V, half leaf removal at véraison; HLR-AV, half leaf removal after véraison; LM-V, leaf moving at véraison; LM-AV, leaf moving after véraison; C, control group.

Sunlight exposure treatments were carried out as described by Matus et al. (2009), with some modifications. In three consecutive years, eight fruit-zone light exposure levels were established in the vines through artificial leaf removal, half leaf removal, or leaf moving (**Figure 1**): leaf removal at berry pepper-corn size (LR-PS); leaf removal at véraison (LR-V); leaf removal after véraison (LR-AV); half leaf removal at véraison (HLR-V); half leaf removal after véraison (HLR-AV); leaf moving at véraison (LM-V); leaf moving after véraison (LM-AV); and non-treated control (C). Leaf removal and half leaf removal treatments were carried out by removing the first one to six basal leaves from the main shoots with clusters and three basal leaves from the first, third, and fifth of each shoot with clusters, respectively. For leaf moving treatment, the first one to six basal leaves of each shoot with clusters were moved aside by the use of nylon zip-ties, in order to increase the sunlight exposure of grape clusters without affecting the photosynthetic carbon assimilation to the fruit. Each treatment was arranged in a completely randomized experimental design with three biological replicates. In each biological replicate, treatment was applied to 15 vines randomly selected from the vineyard’s south and north sites.

The meteorological data during berry development in 2011, 2012, and 2013, including sunlight duration (h), growing degree days (°C), temperature (°C), rainfall (mm) and relative humidity (%), were gathered from the local meteorological administration (Supplementary Table S1). To determine the influence of sunlight exposure on canopy microclimatic conditions, photosynthetically active radiation (PAR) sensor (model S-LIA-M003, Onset Computer Corporation, Bourne, MA, USA) and total radiation sensor (model S-LIB-M003, Onset Computer Corporation, Bourne, MA, USA) were positioned parallel with the cordon at the bunch zone on the defoliated side (west) of the canopy of both exposure and control groups during grape berry development in 2012 and 2013. The air temperature and relative humidity (RH) inside the canopy of each group were also monitored via a Hobo temp/RH smart sensor (model S-THB-M002, Onset Computer Corporation, Bourne, MA, USA) placed at the fruit zone. Each measurement was performed at 5-min intervals (Supplementary Table S2).

Berries from each treatment and control group were sampled at the following developmental time points: 3 weeks after flowering (waf) (berry pepper-corn size; E-L 29), five waf (berry pea-size, E-L 31), seven waf (berry still hard and green, E-L 33), early-véraison (berries begin to color, E-L 35), mid-ripening stage (berries with intermediate Brix values, E-L 36), end of véraison (berries not quite ripe, E-L 37) and complete ripening stage (E-L 38) (Coombe, 1995). For each biological replicate, 600 berries were randomly separated from both sunny and shade sides of at least 100 clusters within 15 vines. The sampling time was fixed at 10:00 to 11:00 am, and three biological replicates were collected with the same method at each sampling date. After being washed with distilled water, a sub-sample of 100 berries from each biological replicate was subjected to the physiological measurements, including berry fresh weight, total soluble solids (TSS) content and titratable acidity (TA), the rest were frozen in liquid nitrogen immediately and transported to the lab in dry ice for the subsequent metabolites determination or transcriptional analysis. TSS concentrations of the juices were measured with digital pocket handheld refractometer (Digital Hand-held Pocket Refractometer PAL-1, Atago, Tokyo, Japan), and TA was determined by titration with NaOH to the end point of pH 8.2 and expressed as tartaric acid equivalent (Cheng et al., 2014).

### Isolation and Identification of Compounds

Phenolic acids were extracted from berries and analyzed as described by Song et al. (2013, in Chinese with English abstract). In detail, a sub-sample of 100 frozen berries randomly selected from each biological replicate was first ground into powder under liquid nitrogen after weighing and removing the seeds. For the analysis of monomeric phenolics, 5 g of ground powder was extracted with 25 mL of 1% (v/v) ascorbic acid and 10 mM EDTA in 4 M NaOH. The extraction mixture was then sonicated for 3 min and shaken in incubator shakers in dark for 8 h under a nitrogen atmosphere at 35°C. After acidification to pH 2 using 6 M HCl and centrifugation at 8,000 rpm for 20 min, the clear supernatant was extracted four times with diethyl ether to obtain the free phenolic acids released from the soluble ester. The combined supernatant was evaporated to dryness, dispersed in 0.5 mL of methanol, and filtered through a 0.45 μm Millipore membrane filter (Millipore Co. Ltd, Billerica, MA, USA) prior to high performance liquid chromatography (HPLC) analysis.

Skin flavonoids were extracted from a sub-sample of 100 berries randomly selected from each biological replicate as described by Li et al. (2014), with some modifications. For flavan-3-ols preparation, 0.1 g sub-sample of skin powder was extracted in a solution of 50 g/L phloroglucinol (1 mL) containing 0.3 N HCl and 0.5% (v/v) ascorbic acid in darkness at 50°C for 20 min. After terminating the reaction by addition of 1 ml NaAc (50 mM), the extraction mixture was centrifuged at 10,000 rpm for 15 min, and the clear supernatant was collected. The residues were re-extracted three times, and all the supernatants were mixed and stored at -40°C. To extract anthocyanins, 0.5 g sub-sample of skin powder was extracted in 10 mL of methanol solution containing 1% formic acid under sonication for 10 min at room temperature, and then shaken in incubator shakers in dark at 25°C for 30 min at a rate of 200 rpm. The extraction mixture was centrifuged at 8,000 rpm for 20 min and the clear supernatant was collected. The residues were re-extracted four times, and all the supernatants were pooled and evaporated to dryness in a rotary evaporator at 30°C, and then dissolved in 10 mL of 10.8% (v/v) acetonitrile aqueous solution with 2% formic acid. All the extracts obtained above were filtered through 0.22 μm nylon membrane filters before HPLC analysis. For flavonols extraction, 5 g sub-sample of skin powder was immersed in 15 mL of 50% ethanol solution containing 1% acetic acid with the aid of ultrasonic vibrations for 35 min at room temperature and then centrifuged at 8,000 rpm for 10 min. The residues were re-extracted four times, and the pooled supernatants were macerated with 50 mL of distilled water and then extracted in 40 mL of ethyl acetate three times. The organic phase was collected and evaporated to dryness in a rotary evaporator at 30°C, and then suspended in 2 mL of 25% methanol. Three independent extractions from three biological repeats were conducted for either the berry or the skin of each sample.

Phenolic acids were monitored on an Agilent 1100 series HPLC-MSD trap VL (Agilent, Santa Clara, CA, USA), equipped with a diode array detector (DAD) and a reversed phase column (Zorbax SB-C18, 250 × 4 mm, 5 μm). The injection volumes were 10 μL and the column thermostat was set at 30°C. Mobile phase A consisted of methanol/acetic acid/water (10:2:88, v/v/v), and mobile phase B consisted of methanol/acetic acid/water (900:15:85, v/v/v). The gradient was from 0 to 3.6% B for 7 min, from 3.6 to 15% B for 19 min, from 15 to 25.5% B for 6 min, from 25.5 to 29.7% B for 3 min, from 29.7 to 45.5% B for 10 min, from 45.5 to 0% B for 8 min, at a flow rate of 1 mL/min. All phenolic acid compounds were identified by matching the retention time and their spectral characteristics against those of standards (Sigma, St. Louis, MO, USA). Chlorogenic acid and caffeic acid were quantified at 325 nm while *p*-coumaric acid, ferulic acid and sinapic acid at 275 nm. The conditions for the mass spectrometry (MS) were as follows: electrospray ionization (ESI) interface; negative ion model; nebulizer pressure, 241.3 kPa; dry gas flow rate, 10 L/min; dry gas temperature, 350°C; Trap ion charge control (ICC), 30,000 units; collision-induced dissociation (CID) voltage, 1.00 V; scan at *m*/*z* 100–1,000.

Qualitative and quantitative analyses of flavonoids were carried out on an Agilent 1200 series HPLC-MSD trap VL linked simultaneously to a DAD (for flavan-3-ols and anthocyanins) or a variable wavelength detector (for flavonols) as described previously (Cheng et al., 2014; Li et al., 2014; Zhu et al., 2014). Flavan-3-ols, anthocyanins and their derivatives were analyzed as in Cheng et al. (2014) and Li et al. (2014), respectively. Flavonols and their derivatives were eluted by using a selection of reverse phase column (Zorbax SB-C18, 50 × 3 mm, 1.8 μm) and binary gradient elution with mobile phase A consisted of acetonitrile/formic acid/water (50:85:865, v/v/v), and mobile phase B consisted of acetonitrile/methanol/formic acid/water (250:450:85:215, v/v/v/v), which was in accordance with Zhu et al. (2014, in Chinese with English abstract) with minor revision. Proportions of solvent B varied as follows: from 0 to 14.2% for 24.2 min, from 14.2 to 15.7% for 2.8 min, from 15.7 to 18.8% for 6.4 min, from 18.8 to 23.5% for 5.4 min, from 23.5 to 26% for 6 min, from 26 to 27.4% for 2 min, from 27.4 to 32% for 4.6 min, from 32 to 40% for 10.2 min, from 40 to 100% for 6 min, from 100 to 0% B for 10.6 min, at a flow rate of 1 mL/min. The injection volumes were 50 μL and the column thermostat was set at 40°C. All flavonol compounds were identified by the UV spectrum and retention time of quercetin-3-*O*-glucoside (Sigma, St. Louis, MO, USA). The detector wavelength was 360 nm. The ESI parameters were as follows: negative ion model, nebulizer pressure, 30 psi; dry gas flow rate, 10 mL/min; dry gas temperature, 325°C; Trap ICC, 30,000 units; CID voltage, 1.00 V; scan at *m*/*z* 100–1,000. Quantitative determination of flavonoids was performed using the external standard method with commercial standards. All analyses were run in replicate and averaged for each biological replicate. One-way ANOVA followed by the Duncan’s new multiple range test was performed using SPSS 20.0 for windows (SPSS Inc., Chicago, IL, USA) to determine significant differences of the physicochemical indexes and phenolic accumulations among treatments at each sampling time point.

### RNA Isolation, Sequencing, and Data Analysis

Based on biochemical parameters and metabolite profiles, we selected the berries of three developmental stages (E-L 36, 37, and 38) from the LR-V and LM-V treatments and the control group during the 2012 growing season to conduct the transcriptome profiling analysis. A sub-sample of 50 berries were randomly selected from each biological replicate for RNA extraction. Total RNAs for RNA-seq analysis were isolated from frozen deseeded berries using a Plant Total RNA Extraction Kit (Sigma, St. Louis, MO, USA), and further purified by DNase I (Promega, Madison, WI, USA) digestion. RNA integrity and concentration were analyzed using the Nanodrop 2000 spectrophotometer (Thermo Fisher Scientific Inc., Wilmington, DE, USA) and the Aglient 2100 Bioanalyzer (Agilent, Santa Clara, CA, USA). Following quality assessment, cDNA libraries constructed from three biological replicates of each sample were sequenced by Illumina Hiseq^TM^2000 sequencer (Illumina Inc., San Diego, CA, USA) with a 50-bp single read module RNA-seq reads and then aligned against the reference grapevine genome V2^1^ using the alignment software Bowtie (Langmead et al., 2009), allowing no more than two nucleotides mismatched. The FPKM (expected fragments per kilobase of transcript per million fragments mapped) method was used for calculating the transcript abundance of each gene (Trapnell et al., 2010). Transcripts were mapped to reference canonical pathways in the Kyoto Encyclopedia of Genes and Genomes (KEGG)^2^ as described previously (Sun et al., 2015a). Prediction of TFs was performed by using the HMMsearch program. Identification of differentially expressed genes (DEGs) between samples was performed with R package ‘NOISeq’ (Tarazona et al., 2011). A threshold of fold-change ≥ 2 and divergence probability ≥ 0.8 was used for filtering the significance of the gene expression difference. Heatmap visualizations were performed using the R package ‘pheatmap’ (Kolde, 2012). Pearson correlation evaluation was conducted with R package ‘Hmisc’ using the rcorr function (Harrell and Dupont, 2012) and co-expression networks were visualized with the Cytoscape software version 3.2.0 (Shannon et al., 2003).

### Quantitative Real-time PCR

Validation of the transcript quantification from the RNA-seq data was carried out through quantitative real-time PCR (qRT-PCR). For extraction of skin RNAs, berry skins from another sub-sample of 50 berries randomly selected from each biological replicate were manually separated from pulps. Total RNA from berry skins was isolated using the same method mentioned above. The subsequent cDNA synthesis and qRT-PCRs were performed as described by Sun et al. (2015a). Gene-specific primers used for qRT-PCR are listed in Supplementary Table S3 (Downey et al., 2003; Castellarin et al., 2006; Fujita et al., 2006; Reid et al., 2006; Bogs et al., 2007; Czemmel et al., 2009; Shimazaki et al., 2011; Azuma et al., 2012; Sun et al., 2015b, 2016). All reactions were run in triplicate, and the normalized relative expression levels of target genes were calculated by 2^-δCt^ (ΔCt = Ct_Target_ – Ct_Control_, Ct: cycle threshold). *VviUbiquitin1* and *Vviβ-Actin* genes were selected as endogenous controls for normalization and Ct_Control_ was the geometric mean of their threshold cycles.

## Results and Discussion

### Berry Development and Ripening

Changes in berry fresh weight, TSS and TA of Cabernet Sauvignon grape berries collected from E-L 31 stage until harvest during the first growing season (2011) and from E-L 29 stage until harvest during the subsequent two growing seasons (2012 and 2013) are shown in Supplementary Figure S1. The date of véraison and harvest were set at approximately eight and sixteen waf, respectively, during all three seasons. Previous studies on several grape species and cultivars reported little or no effect of leaf removal treatments on fruit weights and juice soluble solid contents at harvest (Haselgrove et al., 2000; Main and Morris, 2004; Chorti et al., 2010; Kotseridis et al., 2012). In this study, weight of berries for LR-PS was significantly lower than that of the control at harvest in the 2011 season, while an opposite result was observed in the 2012 season. LM-V and LM-AV significantly increased berry weights from 1 week after each treatment until berry ripening during the 2011 and 2012 seasons, however, there were no discernible differences between each of the two treatments and the control during the third experimental season (Supplementary Table S4). Overall, there were no consistent trends in the differences of berry fresh weight between each of the light-exposure treatment group and the control group during berry development in all the three experimental seasons. Similarly, the differences in the level of juice soluble solids between the exposed and shaded berries during berry development were also inconsistent among different seasons. No treatment differences were found in soluble solid contents at harvest in all the 3 years, except for those of berries from LR-PS, which was higher than that of the control in the 2011 season (Supplementary Figure S1). Specifically, ripening berries from the control and all light-exposure treated clusters have lower soluble solid contents in the 2013 season compared with the other two seasons, which could be attributed to the relative lower level of flowering-to-harvest growing degree days (GDD) accumulation in 2013 than those in the other 2 years (Supplementary Table S1), similar with the results from a previous study (Spayd et al., 2002). The decrease of the TA in the berries from LR-PS treatment during véraison was slightly faster than that of the control during the 2011 and 2012 seasons. However, the influence of LR-PS treatment on TA was negligible during the 2013 season. At harvest, there was no significant difference in the TA among treatments in all the three seasons (Supplementary Figure S1), which was inconsistent with previous studies that leaf removal treatments reduced TA (Zoecklein et al., 1992; Percival et al., 1994), suggesting that TA was differently affected by sunlight exposure depending on cultivar and climate.

### Influence of Cluster Sunlight Exposure Treatments on Phenolic Concentration and Composition

**FIGURE 2:**
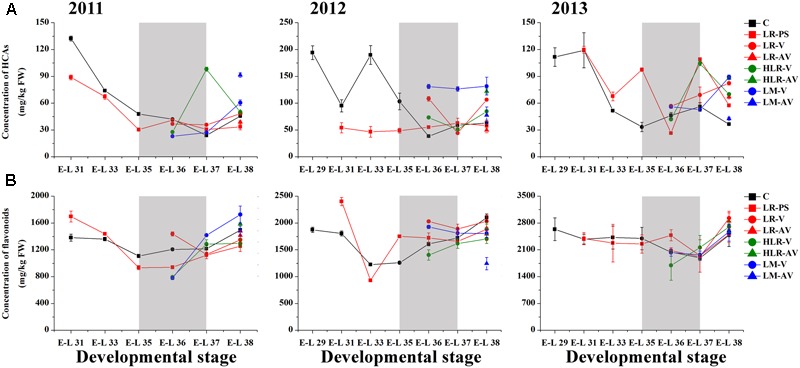
**Changes of the accumulation of (A)** hydroxycinnamic acids (HCA) and **(B)** total flavonoids in different cluster sunlight exposed grape berries during development over three seasons. C, control group; LR-PS, leaf removal at berry pea-size; LR-V, leaf removal at véraison; LR-AV, leaf removal after véraison; HLR-V, half leaf removal at véraison; HLR-AV, half leaf removal after véraison; LM-V, leaf moving at véraison; LM-AV, leaf moving after véraison. Data are mean ± SD of three biological replicates. Light gray background represents the phenological phase of véraison from 5 to 100% of colored berries.

**FIGURE 3:**
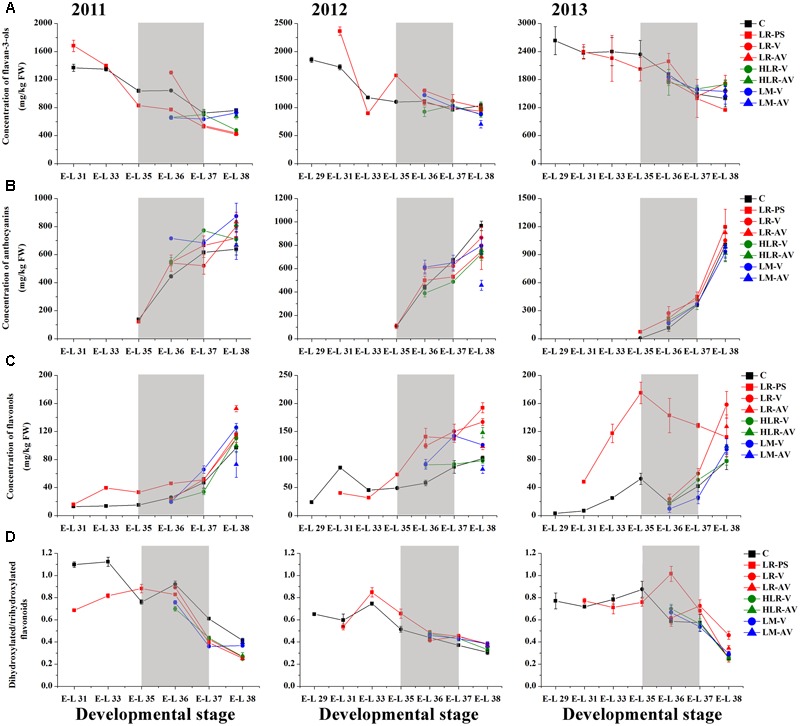
**Changes of the accumulation of (A)** flavan-3-ols, **(B)** anthocyanins, **(C)** flavonols, and **(D)** the ratio of dihydroxylated/trihydroxylated flavonoids in different cluster sunlight exposed grape berry skins during development over three seasons. C, control group; LR-PS, leaf removal at berry pea-size; LR-V, leaf removal at véraison; LR-AV, leaf removal after véraison; HLR-V, half leaf removal at véraison; HLR-AV, half leaf removal after véraison; LM-V, leaf moving at véraison; LM-AV, leaf moving after véraison. Data are mean ± SD of three biological replicates. Light gray background represents the phenological phase of véraison from 5 to 100% of colored berries.

Of the many environmental factors that affect the phenolic biosynthesis in many plants, light has been regarded as one of the major influences (Downey et al., 2004; Cominelli et al., 2008; Wang et al., 2012; Jaakola, 2013). The present study shows that the concentration of total HCAs was significantly increased in the sunlight exposure berries in comparison with the control berries at both véraison and harvest in all the three experimental seasons, except for berries from LR-PS and LR-AV treatments, in which the level of HCAs was slightly higher during véraison while lower at the harvest stage compared with the control in both the 2011 and 2012 seasons (**Figure 2A** and Supplementary Table S4). The results obtained in our study are inconsistent with a previous study conducted on Pinot Noir, which showed that the concentration of HCAs throughout maturation was effectively enhanced by leaf removal at berry set but slight influenced by leaf removal at véraison (Lemut et al., 2011). Of the three classes of flavonoids, flavan-3-ols are present in the greatest proportion in grapes, followed by anthocyanins, with flavonols being present at relatively low levels (Downey et al., 2004). Sunlight exposure did not consistently affect the accumulation of total flavonoids or flavan-3-ols in the skins throughout berry development or at harvest among different seasons (**Figures 2B, 3A**). The concentration of total flavonoids and flavan-3-ols in the skins was slightly lower in LR-PS, LR-V, LR-AV, and HLR-V, while the former was higher in HLR-AV and LM-V treated berries than those from control berries at harvest in the 2011 season. Each of the seven sunlight exposure treatments resulted in decrease in concentration of skin total flavonoids as well as flavan-3-ols at harvest in the 2012 season, while an opposite result was observed in the 2013 season, except for berries from LR-PS, in which the concentration of total flavonoids and flavan-3-ols showed a significant decrease and no discernible difference compared with the control, respectively (Supplementary Table S4). The seasonal variations in the level of flavan-3-ol compounds might be caused by differences of temperature, GDD or the level of PAR during berry ripening among seasons (Supplementary Tables S1, S2), which suggests an ambiguous effect of light-exposure on the flavan-3-ol biosynthesis in grape berry skins.

Anthocyanin biosynthesis has been found to be variably regulated in response to light conditions in a number of sunlight exposure studies, which is possibly confounded by varying experimental settings and other factors, such as cultivar, vineyard location, timing of leaf removal, and growing season. For instance, leaf removal at berry set increased skin anthocyanins in grapes of Merlot, Cabernet Sauvignon and Pinot noir (Lemut et al., 2011; Kotseridis et al., 2012), and pre-bloom leaf removal also substantially increased anthocyanin concentration in Barbera, Lambrusco salamino, Graciano, and Carignan grapes when compared with no leaf removal (Poni et al., 2009; Tardaguila et al., 2010). However, another exposure study indicated that no significant differences among treatments were observed in anthocyanin levels in Nebbiolo grapes at harvest, although leaf removal caused a temporary acceleration of anthocyanin accumulation throughout ripening (Chorti et al., 2010). In the present study, sunlight exposure increased the level of skin anthocyanins at the initiation of grape coloration compared to the control in at least two of the three experimental seasons, while no consistent differences among treatments were observed in the concentration of anthocyanins at harvest over the three experimental seasons (**Figure 3B**). In the 2011 season, almost all exposure treatments could significantly (LR-V, LR-AV, HLR-AV, and LM-V) or slightly (LR-PS and HLR-V) increase skin anthocyanin amount at harvest, whereas no significant differences in the concentration of skin anthocyanins at harvest among treatments were observed in the 2013 season. On the contrary, slight or significant decreases in the skin anthocyanin amount were observed from all of the light exposed berries in the 2012 season (Supplementary Table S4). The diametrically opposed result was possibly due to increased levels of PAR, solar radiation and average temperature for exposed berries during coloration and the maturation phase (Supplementary Table S2), accompanied with a relative high temperature during berry ripening in this experimental season (Supplementary Table S1), which could lead to the inhibit formation or induce degradation of anthocyanins (Yamane et al., 2006; Tarara et al., 2008; Pastore et al., 2013).

Among the three major classes of flavonoid compounds, the accumulation of flavonols was most drastically affected in berry skins under the sunlight exposure treatments. Flavonols have been found to accumulate in sun-exposed tissue of grapes and are thought to act as UV protectants and free radical scavengers (Price et al., 1995; Downey et al., 2004). Leaf removal at all three phenological stages resulted in a dramatic increase in flavonol concentration in the grape skin throughout berry development during the three experimental seasons, similar to the results conducted on Sangiovese berries previously (Pastore et al., 2013), but the degree of their effect was variable among seasons in our experiments (**Figure 3C**). The level of flavonol compounds was also moderately increased by HLR-AV and LM-V treatments, except that there was no significant difference between the control and HLR-AV in the 2011 season. HLR-V and LM-AV did not significantly influence the concentration of skin flavonols at harvest compared with the control, although there was a temporary acceleration of flavonol accumulation throughout ripening in HLR-V treated berries (**Figure 3C** and Supplementary Table S4). However, opposite results regarding changes in flavonol contents under different sunlight exposure treatments have been found previously, in which leaf moving at véraison increased flavonol synthesis greater than leaf removal treatment (Matus et al., 2009), suggesting that the enhanced accumulation of flavonols under treatments is strongly associated with the severity degree of sunlight exposure, and also climate divergences in different years and regions.

In the flavonoid biosynthesis pathway, two metabolic branches leading to the biosynthesis of B-ring dihydroxylated (3,3′-OH) and trihydroxylated (3,3′,5′-OH) subunits were reported to have different sensitivities in response to various lighting conditions in many previous researches (Downey et al., 2004; Azuma et al., 2012; Guan et al., 2015). Our results showed that the molar ratio of dihydroxylated to trihydroxylated flavonoids was continuously decreased throughout berry ripening, while changes of the ratio among treatments were inconsistent during the three seasons (**Figure 3D**). In the first experimental season, the ratio of dihydroxylated/trihydroxylated flavonoids in berries from almost all of the sunlight exposure treatments was decreased compared with the control across development, except for LR-PS at E-L 33 stage, LR-V at E-L 35 stage and LM-AV at harvest. In contrast, however, light-exposure caused a slight or marked increase in the ratio of dihydroxylated/trihydroxylated flavonoids during berry ripening in the 2012 growing season. In the last experimental season, no significant differences in the ratio of dihydroxylated/trihydroxylated flavonoids were observed among treatments except for berries from the leaf removal treatments, which was increase in LR-V and LR-AV treated berries at harvest and in LR-PS treated berries during coloration (**Figure 3D** and Supplementary Table S4). In contrast to the results obtained from cluster shading studies (Koyama and Goto-Yamamoto, 2008; Guan et al., 2015), the effect of light-exposure was possibly influenced by the temperature or other climate variables of the year.

### Light-induced Transcriptional Changes of Phenolic Biosynthetic Pathway Genes

**FIGURE 4:**
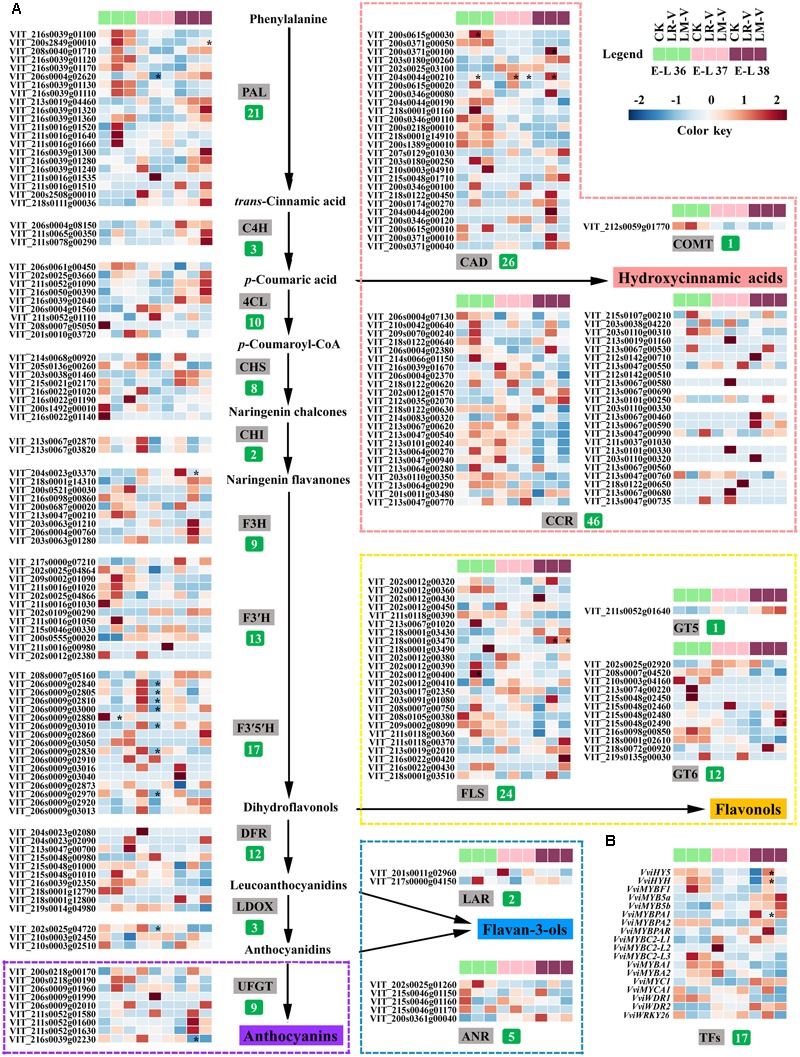
**Effects of sunlight exposure on the transcript profile of the (A)** enzymes and **(B)** regulatory factors involved in phenolic biosynthesis in grape berries. PAL, phenylalanine ammonia-lyase; C4H, *trans*-cinnamate 4-monooxygenase; CCR, cinnamoyl-CoA reductase; CAD, cinnamyl-alcohol dehydrogenase; COMT, caffeic acid 3-*O*-methyltransferase; 4CL, 4-coumarate: CoA ligase; CHS:,chalcone synthase; CHI, chalcone isomerase; F3H, flavanone 3-hydroxylase; F3′H, flavonoid 3′-hydroxylase; F3′5′H: flavonoid 3′,5′-hydroxylase; FLS, flavonol synthase; GT5, uridine diphosphate (UDP)-glucuronic acid:flavonol-3-*O*-glucuronosyltransferase; GT6, bifunctional UDP-glucose/UDP-galactose:flavonol-3-*O*-glucosyltransferase/galactosyltransferase; DFR, dihydroflavonol reductase; LAR, leucoanthocyanidin reductase; LDOX, leucoanthocyanidin dioxygenase; ANR, anthocyanidin reductase; UFGT, UDP-glucose:flavonoid 3-*O*-glucosyltransferase. The number of expressed family members for each enzyme is indicated in green box. Each square in the heatmap located beside their gene names corresponds to the average FPKM value of the gene in each sample as illustrated in the legend. Genes with significant expression changes compared with the control groups in each developmental stage are indicated by asterisks (^∗^) in the squares. Expression profiles of specific structural genes required for the biosynthesis of HCAs, flavonols, flavan-3-ols and anthocyanins are shown in pink, yellow, and violet dotted boxes, respectively. C, control group; LR-V, leaf removal at véraison; LM-V, leaf moving at véraison.

To investigate the responses of phenylpropanoid/flavonoid biosynthetic pathway related structural and regulatory genes to different light-exposure treatments, berries of three distinct development stages (E-L 36, 37, and 38) from the LR-V and LM-V treated and the control groups during the 2012 growing season were selected to characterize the changes in gene expression at the transcript level by RNA-seq. Results showed that the general structural genes of phenylpropanoid and flavonoid metabolic pathways, including some members of phenylalanine ammonia-lyase (PAL, EC 4.3.1.24), 4-coumarate: CoA ligase (4CL, EC 6.2.1.12) and flavanone 3-hydroxylase (F3H, EC 1.14.11.9), were significantly or moderately (fold-change ≥ 2 while divergence probability ≤ 0.8) up-regulated in berries from the LR-V and LM-V treatment groups at E-L 36 and 38 stages while down-regulated at E-L 37 stage. Furthermore, almost all members of CHS (EC 2.3.1.74) and chalcone isomerase (CHI, EC 5.5.1.6) were moderately down-regulated in sunlight exposed grapes in comparison with those from the control group across the three developmental stages. The expression of members of specific structural genes required for HCA and flavonol biosynthesis, including cinnamyl-alcohol dehydrogenase (CAD, EC 1.1.1.195) and FLS (EC 1.14.11.23) across the three developmental stages as well as bifunctional UDP-glucose/UDP-galactose:flavonol-3-*O*-glucosyltransferase/galactosyltransferase (GT6, EC 2.1.1.76) at E-L 38 stage, and members of dihydroflavonol reductase (DFR, EC 1.1.1.219) and UFGT (EC 2.4.1.115) required for anthocyanin biosynthesis at E-L 36 and 37 stages were significantly or moderately up-regulated in LR-V and LM-V treated berries, leading to the increased accumulation of corresponding phenolic products. The lower contents of flavan-3-ols in LR-V and LM-V treated berry skins compared with the control group at E-L 37 and 38 stages is supported by the moderately down-regulation of members of leucoanthocyanidin reductase (LAR, EC 1.17.1.3) and anthocyanidin reductase (ANR, EC 1.3.1.77), which are directly involved in flavan-3-ol biosynthesis (**Figures 3, 4A**).

It was previously reported that *VviFLS4* was the sole member of the grapevine *FLS* family which specifically responded to different light regimes and showed a clear expression pattern corresponding to the accumulation of flavonols in the berry skins (Fujita et al., 2006; Matus et al., 2009; Koyama et al., 2012; Pastore et al., 2013). In our study, the transcription of several other members of *FLS* family in addition to *VviFLS4* (VIT_218s0001g03470) was also drastically induced (fold-change ≥ 2) by LR-V and/or LM-V treatments in different developmental stages, such as VIT_202s0012g00390, VIT_202s0012g00400 and VIT_202s0012g00450 in LR-V treated berries at E-L 36 stage, as well as VIT_208s0007g00750 and VIT_213s0067g01020 in LR-V and LM-V treated berries at both E-L 36 and 38 stages (**Figure 4A**). These results were consistent with the increased accumulation of flavonols in light-exposure berries, which indicates that grapevine *FLS* gene family may be functionally redundant in response to light signal. The hydroxylation pattern of flavonoids is known to be mediated by the enzyme activity of F3′H (EC 1.14.13.21) and F3′5′H (EC 1.14.13.88), which catalyze the hydroxylation of naringenin and dihydrokaempferol at the 3′ and 3′5′ positions of the B-ring, respectively (Bogs et al., 2006; Guan et al., 2015). Our results showed that no significant differences in the expression level of genes encoding F3′H among treatments were detected at each sampling point, but the transcription abundance of several *F3*′*5*′*H* family members was significantly down-regulated by LR-V and moderately down-regulated by LM-V at both E-L 36 and 37 stages (**Figure 4A**), which were in fair agreement with the higher ratio of dihydroxylated/trihydroxylated flavonoids observed in berries from LR-V and LM-V treatments, in comparison with those in berries from the control group (**Figure 3D**).

To identify additional genes that might contribute to alterations in phenolic metabolism in berries grown under different light conditions, the transcription profile of phenolic biosynthesis-related genes was compared with the HCA, total flavonoid, flavonol, flavan-3-ol, and anthocyanin profiles of all samples, respectively. The correlation analysis based on pearson’s coefficient revealed that the expression of four members of PAL (VIT_216s0039g01100, VIT_216s0039g01110, VIT_216s0039g01120, and VIT_216s0039g01130), the first committed enzyme in phenylpropanoid metabolism (Sparvoli et al., 1994), was highly significantly (*p*-value ≤ 0.01) correlated with the accumulation of flavonoids in berries from different light treated groups. The transcript of two other genes (VIT_213s0047g00210 and VIT_206s0061g00450) belonging to the *4CL* and *F3H* families was also significantly (*p*-value ≤ 0.05) correlated with the changes of flavonoid content in each samples. In addition, there was a significant correlation (*p*-value ≤ 0.05) between flavonol accumulation and the expression of two members of *FLS* family (*VviFLS4* and VIT_208s0007g00750) mentioned above (Supplementary Table S5). However, no specific structural genes for phenolic biosynthesis pathway were found to be correlated with the accumulation of HCAs, flavan-3-ols or anthocyanins, which indicates that the biosynthesis or degradation of these compounds in berries under different light regimes might be controlled by the cooperation of multiple enzymes from the entrance to branches.

### Expression Analysis of Flavonoid Biosynthesis-related Transcription Factors

In grapes, some members of R2R3-MYB TF family and their co-activators belonging to other TF families (bHLH and WDR) which could regulate the transcription of downstream target genes required for phenolic biosynthesis pathways have been isolated and characterized recently (Hichri et al., 2011). The expression of these TFs involved in flavonoid metabolism has also been reported to be induced or suppressed by many environmental factors, such as light quality, temperature and water deficit conditions (Castellarin et al., 2007; Cominelli et al., 2008; Azuma et al., 2012). The transcript level of VviMYBF1, which acts as a direct regulator of *VviFLS4* expression (Czemmel et al., 2009; Matus et al., 2009), was moderately greater in berries after light-exposure treatments than those in the control berries, except for a slight decrease in LM-V treated berries at E-L 37 stage compared with the control (**Figure 4B**). Expression of two UV-B-inducible grapevine flavonol synthesis regulators, *VviHY5* and *VviHYH* (Loyola et al., 2016), was also significantly and moderately up-regulated in LR-V and LM-V treated berries at post-véraison berry developmental (E-L 38) stage, respectively. These results were well consistent with an increase in the transcript abundances of members of *FLS* as well as flavonol concentrations after light-exposed treatments, which indicates that light affects flavonol biosynthesis through transcript activation of a series of TFs and structural genes. Several regulators of the general branch and different branches of flavonoid synthesis, including VviMYB5a, VviMYB5b, VviMYBPA1, VviMYBPA2, VviMYBPAR, VviMYBC2-L1, VviMYBC2-L2, VviMYBC2-L3, and a TTG2-like homolog protein VviWRKY26 (Amato et al., 2016), showed large divergent changes in the transcript levels during berry development or under different light-exposure treatments (**Figure 4B**). Transcript abundances for VviMYB5a and VviMYB5b, VviWRKY26, as well as the negative regulator of PA accumulation VviMYBC2-L1 (Huang et al., 2014) in grape berries presented a high level during the three development stages but did not respond to changing light conditions, while low levels of *VviMYBPA2, VviMYBPAR, VviMYBC2-L2*, and *VviMYBC2-L3* transcripts were detected in berries from all treatment groups. In addition, the expression of *VviMYBPA1* was significantly and slightly down-regulated in LR-V and LM-V treated berries at E-L 38 stage, respectively, which correlated well with the changes of *LDOX* expression and total flavan-3-ol contents at this stage. Therefore, it may be speculated that the transcript level of VviMYBPA1 might lead to the difference in the flavan-3-ol contents of grape berries growing under different light conditions. Furthermore, transcript abundances of VviMYBA1 and VviMYBA2, two regulators of the anthocyanin branch (Walker et al., 2007), were slightly up-regulated at E-L 36 stage while down-regulated at E-L 37 or 38 stage in light exposed berries, correlating with the anthocyanin levels responded to light conditions in the grape skin. However, no significant changes in the expression of two bHLH factors, VviMYC1 and VviMYCA1, as well as two WDR proteins VviWDR1 and VviWDR2 among different light-exposure groups were observed (**Figure 4B**), although they have been reported to be involved in anthocyanin and/or PA synthesis (Hichri et al., 2010; Matus et al., 2010) and differentially modulated by different light qualities in other plant species (Sompornpailin et al., 2002; Cominelli et al., 2008).

### Validation of RNA-seq by Quantitative Real-time PCR

To validate the expression profiles obtained from the RNA-seq data, 15 genes relating to our biological focus were selected to subject to qRT-PCR analysis. They included 12 phenylpropanoid/flavonoid biosynthetic pathway related structural genes (*VviPAL1, VviPAL2, VviPAL7, VviPAL15, VviF3H1, VviF3H2, VviF3’H, VviF3′5′H, VviFLS1, VviFLS2, VviFLS3*, and *VviFLS4*), as well as VviMYBF1, VviMYBPA1 and VviMYBA1 TF genes involved in the regulation of flavonol, flavan-3-ol and anthocyanin biosynthesis, respectively (Supplementary Figure S2). Two housekeeping genes in *V. vinifera, VviUbiquitin1* and *Vvi*β*-Actin* were used as endogenous controls for normalization as their relatively constant expression throughout grape berry development as well as in berries under various stress conditions (Downey et al., 2003; Reid et al., 2006). The results showed that the expression of 15 genes determined by qRT-PCR was significantly correlation with those from the RNA-seq data at the 0.01 level (*r* = 0.54), thus verifying the method.

### Co-expression Analysis between Metabolic Pathway Genes and Transcription Factor Genes

**FIGURE 5:**
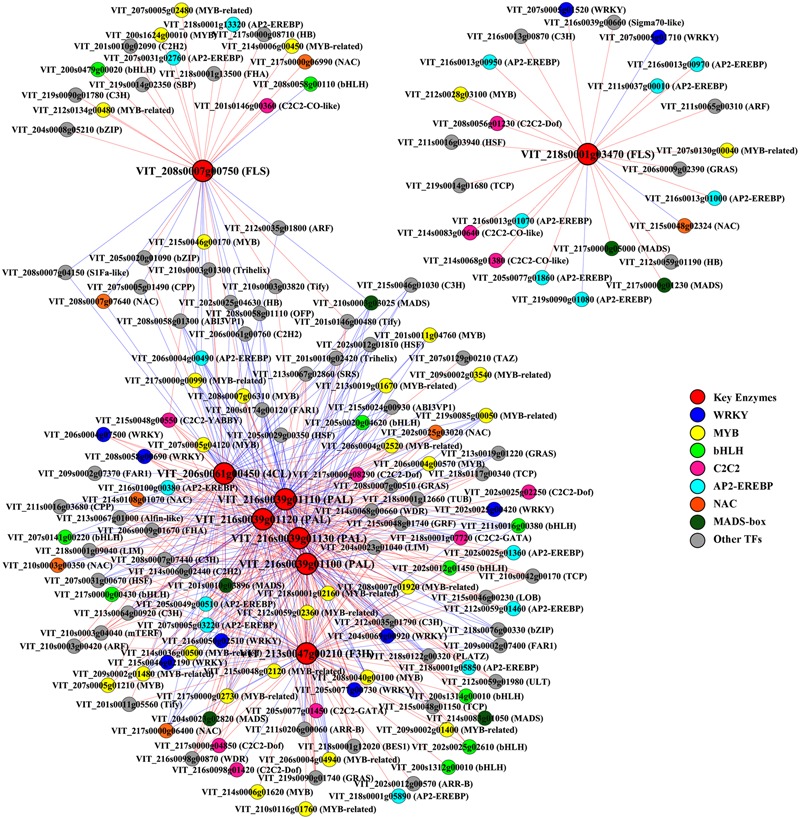
**Co-expression analysis of key enzymes required for phenolic biosynthesis and transcription factors (TFs) in berries from different cluster sunlight exposed grapes during development.** Key enzymes are assigned in *rectangle* and TFs in *ellipse*. PAL, phenylalanine ammonia-lyase; 4CL, 4-coumarate: CoA ligase; F3H, flavanone 3-hydroxylase; FLS, flavonol synthase. Positive and negative correlations are provided by red arrows and blue inhibitory arrows, respectively.

Transcriptome co-expression analysis, which is based on the assumption that genes with similar expression patterns are most likely to be functionally associated, has proven to be a powerful tool for revealing regulatory networks of genes involved in linked processes (Persson et al., 2005). In plants, this strategy has been applied to identify factors regulating several metabolic pathways, such as two *Arabidopsis* MYB TFs regulating aliphatic glucosinolate biosynthesis and a rice AP2/EREBP (APETALA 2/ethylene responsive element binding protein) family TF involved in starch biosynthesis (Hirai et al., 2007; Fu and Xue, 2010). To systemically identify unknown putative regulators that control the phenolic biosynthesis in grape berries in response to different light regimes, a genome-wide co-expression analysis was employed between metabolic pathway genes and TF genes. Eight phenolic synthesis genes screened previously, including genes encoding PALs, 4CL, F3H, and FLSs, were selected as “guide genes” to identify co-expression relationships specific to the light-induced differentially expressed TF genes using expression data of all light treated and control samples from RNA-seq. Any two genes with an absolute value of the Pearson Correlation Coefficient (PCC) greater than 0.7 (or 0.8) and *p*-value less than or equal to 0.05 (or 0.01) between their expression profiles were considered as significant (or highly significant) co-expressed genes (Fu and Xue, 2010). The results showed that a total of 120 and 59 TFs were highly co-expressed with the six total flavonoid biosynthesis-related (group I) and the two flavonol biosynthesis-related (group II) guide genes, respectively (**Figure 5**). Among the identified group I co-expressed TFs, the most abundant positively correlated TFs were members of the MYB, WRKY, C2C2, AP2/EREBP, bHLH, and MADS-box families (*p*-value ≤ 0.01), whereas the most abundant negatively correlated TFs belonging to MYB, NAC (No apical meristem, ATAF 1,2, Cup-shaped cotyledon 2), Cys2/His2 (C2H2) type and CCCH type (C3H) zinc finger protein families (*p*-value ≤ 0.01). Similarly, specific members of MYB, AP2/EREBP and C2C2 families were also found to be the most abundant significantly positively co-expressed TFs with group II (*p*-value ≤ 0.05).

In all plant species analyzed to date, MYB TFs, together with bHLH and WDR proteins, act as common denominators in the regulation of flavonoid accumulation under various biotic or abiotic signals, such as high-light, UV, drought, and extreme temperatures (Koes et al., 2005; Xu et al., 2015). Some additional potential transcriptional regulators that belong to WRKY, MADS-box, and bZIP TF families have also been reported to be involved in specific branches of the phenylpropanoid metabolism (Hichri et al., 2011). Besides, several negative regulators of flavonoid synthesis, such as R2R3-MYB, single domain R3-MYB repressors and truncated bHLH, inhibit the formation of MBW complex or modify it, thereby actively repress transcription in plants (Liu et al., 2015). The positively or negatively co-expression of multiple members of MYB, bHLH, WRKY, and MADS-box TF families with those key genes in the present result suggests that light-regulated flavonoid biosynthesis in grape berries is maintained by a complex regulatory network involves both positive and negative feedback loops. By using over-expressing and antisense transgenic plant strategies, it was shown that some DOF (DNA-binding One Zinc Finger) genes from the C2C2 zinc finger-containing TF superfamily putatively involved in regulation of enzymes of the phenylpropanoid and flavonoid pathways in *Arabidopsis* (Noguero et al., 2013). The plant-specific AP2/EREBP TF family, which was composed of AP2, DREB (*cis*-acting dehydration responsive element-binding protein), RAV (related to ABI3/VP1), ERF (ethylene responsive factor) and other subfamilies, plays a major role in several developmental processes, and also participates in plant hormone signal transduction as well as plant’s responses to pathogens and various environmental stresses (Dietz et al., 2010). Recent studies revealed that a class of repressor-type ERF-subfamily TFs act as active or passive repressors of transcription *via* their ERF-associated amphiphilic repression (EAR) domain, which was also found in some C2H2 type zinc-finger proteins and R2R3-MYB repressors of flavonoid synthesis-related genes in various plant species (Aharoni et al., 2001; Ciftci-Yilmaz and Mittler, 2008; Huang et al., 2014). NAC proteins are another plant-specific TFs which have been shown to play an essential role in regulating senescence, cell division, and wood formation, and also participate in plant response to pathogens, viral infections, and various environmental stresses (Nakashima et al., 2012). It was also reported that a NAC protein in *Arabidopsis*, ANAC078, positively regulates the expression of genes related to the biosynthesis of flavonoids, subsequently leading to the accumulation of anthocyanins in response to high-light (Morishita et al., 2009). Furthermore, VviNAC29, a protein belonging to the grapevine NAC TF superfamily, was demonstrated to act as a cooperative regulator controlling the stress-responsive expression of *VviF3′H* in our previous study (Sun et al., 2015b). However, their roles in the negative regulation of the flavonoid synthesis-related genes have not been investigated previously, thus, the conclusion that whether they could directly or indirectly regulate the light-response of phenolic biosynthesis still needs to be further characterized.

### Light Response of Plant Hormone Signal Transduction Related Genes

**FIGURE 6:**
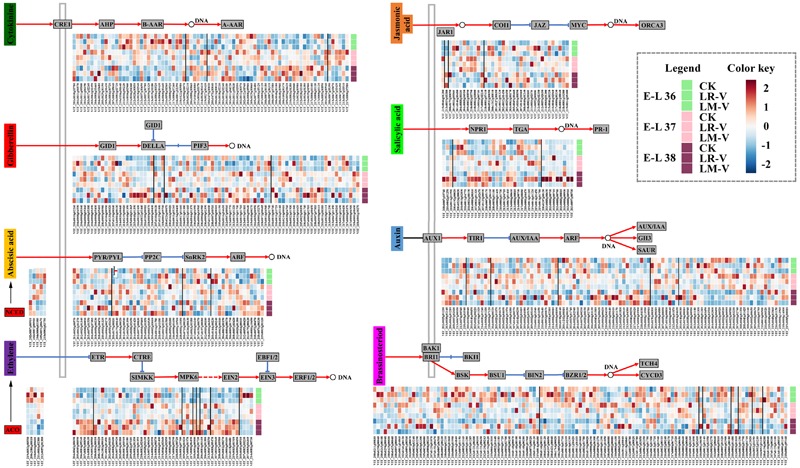
**Effects of sunlight exposure on the expression profile of phytohormone biosynthesis and signaling genes.** CRE1, cytokinin receptor (histidine kinase 2/3/4); AHP, histidine-containing phosphotransfer protein; B-AAR, type-B primary two-component response regulator; A-AAR, type-A primary two-component response regulator; GID1, gibberellin-insensitive dwarf1 (gibberellin receptor); GID2, F-box protein gibberellin-insensitive dwarf2; DELLA, DELLA protein; PIF3, phytochrome-interacting factor 3; NCED, 9-*cis*-epoxycarotenoid dioxygenase; PYR/PYL, abscisic acid receptor PYR/PYL family; PP2C, protein serine/threonine phosphatase 2C; SnRK2, serine/threonine-protein kinase SRK2; ABF, ABA responsive element binding factor; ACO, 1-aminocyclopropane-1- carboxylic acid oxidase; ETR, ethylene receptor; CTR1, serine/threonine-protein kinase CTR1; SIMKK, mitogen-activated protein kinase kinase; MPK6, mitogen-activated protein kinase 6; EIN2, ethylene-insensitive protein 2; EIN3, ethylene-insensitive protein 3; EBF1/2, EIN3-binding F-box protein; ERF1/2, ethylene-responsive TF 1/2; JAR1, jasmonic acid-amino synthetase; COI1, coronatine-insensitive protein 1; JAZ, jasmonate ZIM domain-containing protein; MYC2, bHLH TF MYC2; ORCA3, octadecanoid-derivative responsive Catharanthus APETALA2-domain 3; NPR1, non-expressor of pathogenesis-related genes1; TGA, bZIP TF TGA; PR-1, pathogenesis-related protein 1; AUX1, auxin influx carrier; TIR1, transport inhibitor response 1; AUX/IAA, auxin-responsive protein IAA; ARF, auxin response factor; GH3, indole-3-acetic acid-amido synthetase; SAUR, small auxin-up RNA; BAK1, brassinosteroid insensitive 1-associated receptor kinase 1; BRI1, protein brassinosteroid insensitive 1; BKI1, BRI1 kinase inhibitor 1; BSK, brassinosteroid signaling kinase; BSU1, bri1 suppressor 1; BIN2, protein brassinosteroid insensitive 2; BZR1/2, brassinosteroid resistant 1/2; TCH4, xyloglucan:xyloglucosyl transferase; CYCD3, cyclin D3. Each square in the heatmap located beside their gene names corresponds to the average FPKM value of the gene in each sample as illustrated in the legend. Positive and negative regulations of hormone signalings are indicated by red arrows and blue inhibitory arrows, respectively.

Phytohormones have been implicated in controlling various aspects of grape berry development, in particular, the important processes of ripening and adaptation to adverse environmental conditions, including harmful UV radiation (Jeong et al., 2004). In some cases, hormone pathways act downstream of the light signal pathways to regulate growth, whereas in other cases they interact with each other reciprocally (Alabadí and Blázquez, 2009). In LR-V treated berries, the transcript abundances of some members of PYR/PYL (VIT_208s0058g00470 and VIT_210s0003g01335) and abscisic acid (ABA) responsive element binding factor (ABF; VIT_208s0007g03420) involved in ABA signal transduction (Klingler et al., 2010), xyloglucan:xyloglucosyl transferase TCH4 (VIT_211s0052g01190) involved in BR signal transduction (Clouse, 2015), jasmonate ZIM domain-containing protein (JAZ; VIT_201s0146g00480) and MYC2 TF (VIT_211s0052g00100) involved in jasmonic acid (JA) signal transduction (Fernández-Calvo et al., 2011), as well as TGA TF (VIT_207s0031g02670 and VIT_208s0007g06160) and pathogenesis-related protein 1 (PR-1; VIT_203s0088g00780, VIT_203s0088g00810, VIT_203s0088g00910, and VIT_203s0097g00700) involved in salicylic acid (SA) signal transduction (Eulgem, 2005) were significantly or moderately higher compared with that of the control at nearly all the three developmental stages (**Figure 6**). In addition, the transcription of some proteins involved in other plant hormone signal transductions was also significantly or moderately up-regulated in LR-V treated berries at specific developmental stages, such as members of the histidine kinase receptors CRE1 (VIT_213s0019g01180 and VIT_217s0000g04920) and histidine-containing phosphotransfer protein (AHP; VIT_211s0016g03170) required for cytokinin (CTK) signal transduction (Choi and Hwang, 2007) and gibberellin receptor GID1 (VIT_213s0084g00130) required for Gibberellin (GA) signal transduction (Hirano et al., 2008) at E-L 36 stage, members of the auxin influx carrier AUX1 (VIT_208s0007g02030) and auxin-responsive protein IAA (AUX/IAA; VIT_211s0016g03540) required for auxin signal transduction (Lau et al., 2008) at E-L 38 stage. Notably, a member of serine/threonine-protein kinase CTR1 (VIT_218s0001g07700), a negative regulator of the ethylene (ETH) response pathway (Chen et al., 2005), was up-regulated in LR-V treated berries at E-L 38 stage. Meanwhile, a member of 1-aminocyclopropane-1-carboxylic acid oxidase (ACO, EC 1.14.17.4; VIT_211s0016g02380), the last enzyme in the ETH production pathway which controlled the biosynthesis of ETH in plants (Chervin et al., 2004), was significantly down-regulated in the same developmental stage. In berries from LM-V treatment, a member of TCH4 (VIT_211s0052g01190) was significantly up-regulated, while members of AUX/IAA (VIT_214s0030g02310) and PR-1 (VIT_203s0088g00710) were significantly down-regulated at E-L 37 stage. No significant differences in the expression of plant hormone signal transduction related genes between berries from LM-V treatment and the control group were detected at both E-L 36 and 38 stages. Nevertheless, a member of 9-*cis*-epoxycarotenoid dioxygenase (NCED, EC 1.13.11.51; VIT_219s0093g00550), which limits the level of ABA in the biosynthesis pathway (Zhang et al., 2009), was up-regulated in LM-V treated berries at E-L 36 stage, while a member of ACO (VIT_211s0016g02380) was down-regulated at E-L 38 stage (**Figure 6**).

Previous studies indicate that the accumulation of phenolics in berry skin during the ripening stage, as well as the expression of structural genes and their transcriptional regulators considered to be involved in the phenylpropanoid and flavonoid pathways, were enhanced by exogenous ABA and ETH treatments, while suppressed by synthetic auxins, NAA (Ban et al., 2003; El-Kereamy et al., 2003; Jeong et al., 2004; Fujita et al., 2006; Koyama et al., 2010). The acceleration of berry ripening and flavonoid accumulation in LR-V treated grape berries was well correlated with the enhancement of ABA signal transduction, which might act as a protective mechanism induced by enhanced light irradiation (Berli et al., 2011), while the increased auxin signal transduction and decreased biosynthesis of ETH might result in the suppression of flavonoid biosynthesis, especially flavan-3-ols and anthocyanins at harvest. Moreover, the different expression of metabolic enzymes of phytohormones at E-L 36 and 38 stages, and the transcriptional changes of AUX/IAA at E-L 37 stage, were shown to be perfectly correlated with changes in the accumulation of flavonoids in LM-V treated berries. A number of studies have indicated that light signaling affects the biosynthesis and/or signaling of multiple phytohormones such as auxin, GA, CTKs, ETH, and BRs (Carvalho et al., 2011). The interactions between light and hormones pathways operate through distinct molecular mechanisms in plants and play an important role in the adjustment of developmental programs and behavior of the plants to the environment (Alabadí and Blázquez, 2009). Taken together, our results suggest that phenolic metabolic in berry skins of the Cabernet Sauvignon grape is precisely controlled by a series of phytohormones in response to exchanged light irradiation.

## Conclusion

In the present study, the transcriptional profiles and metabolite profiles of phenolic biosynthesis pathway were analyzed in Cabernet Sauvignon grapes under different sunlight exposure treatments during berry development. Leaf removal or leaf moving at different berry development stages did not show consistent effects on the accumulation of flavan-3-ol, anthocyanin or total flavonoids in grape berries over three seasons. However, the concentrations of HCAs and flavonols were moderately and drastically increased in sunlight exposed grape berries, respectively, which is well correlated with changes in transcriptional abundance of *PAL, 4CL, F3H*, and *FLS* family members as well as large amounts of regulatory genes. Furthermore, the transcriptional changes of genes required for the biosynthesis and signal transduction of auxin, ETH and ABA were found to be exactly in accordance with the accumulation of phenolics in light exposed berries during development, confirmed the importance of phytohormones on berry phenolic biosynthesis of grapes in response to light environment. Taken together, our results provide new valuable insights into understanding of the complex regulatory network of sunlight-responsive phenolic biosynthesis in grape berries, as well as theoretical foundations for cultivation management and wine production.

## Author Contributions

JW and WC conceived and designed the experiments. GC and Y-NH did the field experiments. R-ZS, GC, and QL analyzed the transcriptome sequencing data. GC, QL, YW, Y-RZ, W-FS, XZ, and X-DC performed the HPLC quantification. Y-BL and S-YL provided technical support. R-ZS and JW wrote the paper. All the authors revised it critically for important intellectual content and approved the final version of this manuscript.

## Conflict of Interest Statement

The authors declare that the research was conducted in the absence of any commercial or financial relationships that could be construed as a potential conflict of interest.

## Footnotes

1 http://genomes.cribi.unipd.it/grape/

2 http://www.genome.ad.jp/kegg/
