# Supplementary material for: Light-induced Variation in Phenolic Compounds in Cabernet Sauvignon Grapes (Vitis vinifera L.) Involves Extensive Transcriptome Reprogramming of Biosynthetic Enzymes, Transcription Factors, and Phytohormonal Regulators
Source: Front Plant Sci. 2017 Apr 19;8:547. doi: 10.3389/fpls.2017.00547 (PMC5395571; doi:10.3389/fpls.2017.00547)
Supplement: Supplementary file 1 [file Data_Sheet_1.docx]

Supplementary Material

Light-induced Variation in Phenolic Compounds in Cabernet Sauvignon Grapes (Vitis vinifera L.) Involves Extensive Transcriptome Reprogramming of Biosynthetic Enzymes, Transcription Factors, and Phytohormonal Regulators

Run-Ze Sun, Guo Cheng, Qiang Li, Yan-Nan He, Yu Wang, Yi-Bin Lan, Si-Yu Li, Yan-Rong Zhu, Wen-Feng Song, Xue Zhang, Xiao-Di Cui, Wu Chen, Jun Wang*

*** Correspondence:** Dr. Jun Wang: jun_wang@cau.edu.cn

# Supplementary Data

Supplementary Material should be uploaded separately on submission. Please include any supplementary data, figures and/or tables.

Supplementary material is not typeset so please ensure that all information is clearly presented, the appropriate caption is included in the file and not in the manuscript, and that the style conforms to the rest of the article.

# Supplementary Figures and Tables

For more information on Supplementary Material and for details on the different file types accepted, please see [here](http://home.frontiersin.org/about/author-guidelines#SupplementaryMaterial).

## Supplementary Figures


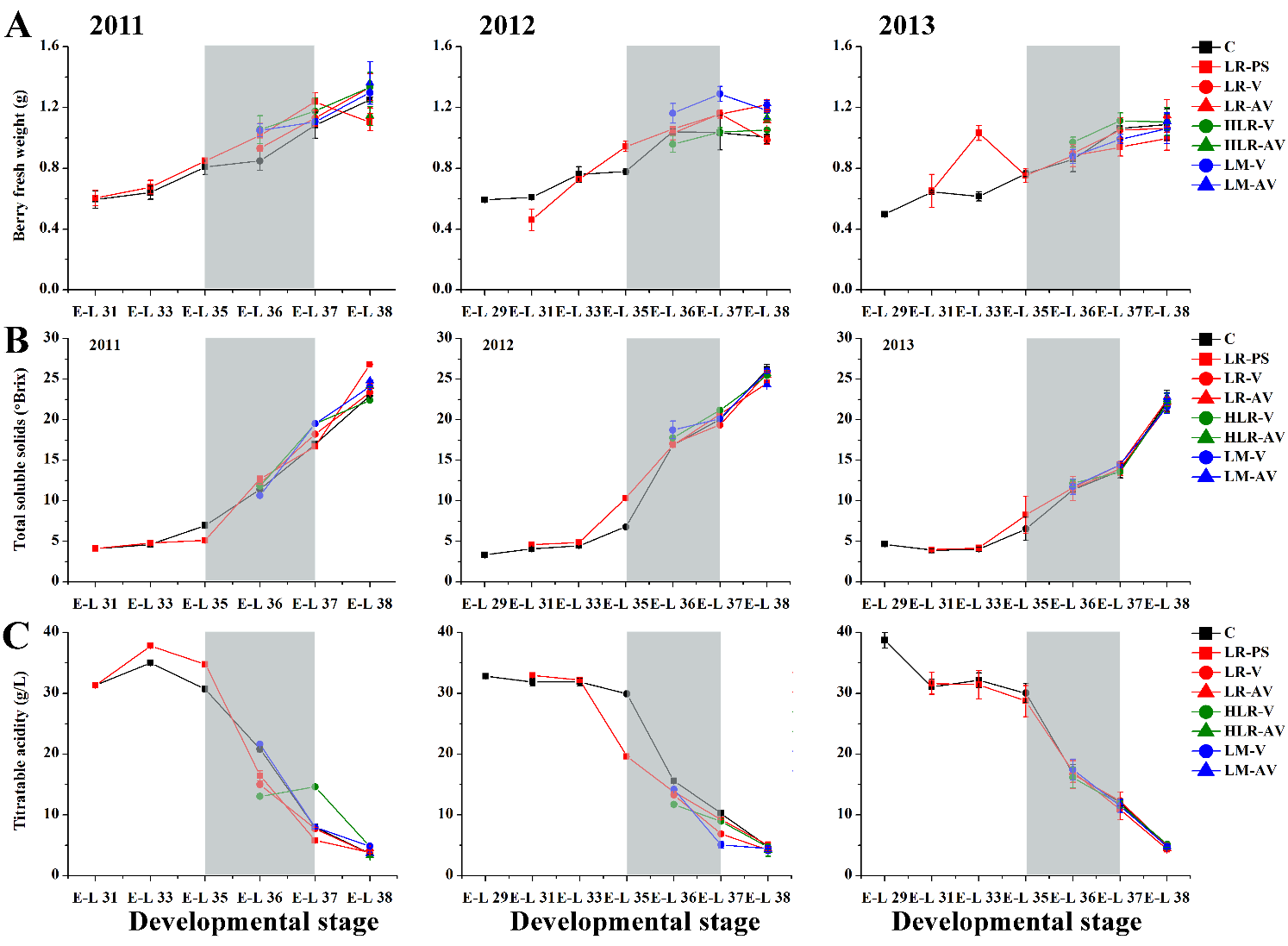


**Figure S1.** Influence of cluster sunlight exposure treatments on (A) fresh weight, (B) total soluble solids and (C) titratable acidity contents of Cabernet Sauvignon grape berries during ripening over three seasons. C: control group; LR-PS: leaf removal at berry pea-size; LR-V: leaf removal at véraison; LR-AV: leaf removal after véraison; HLR-V: half leaf removal at véraison; HLR-AV: half leaf removal after véraison; LM-V: leaf moving at véraison; LM-AV: leaf moving after véraison. Data are mean ± SD of three biological replicates. Light grey background represents the phenological phase of véraison from 5% to 100% of coloured berries.

##
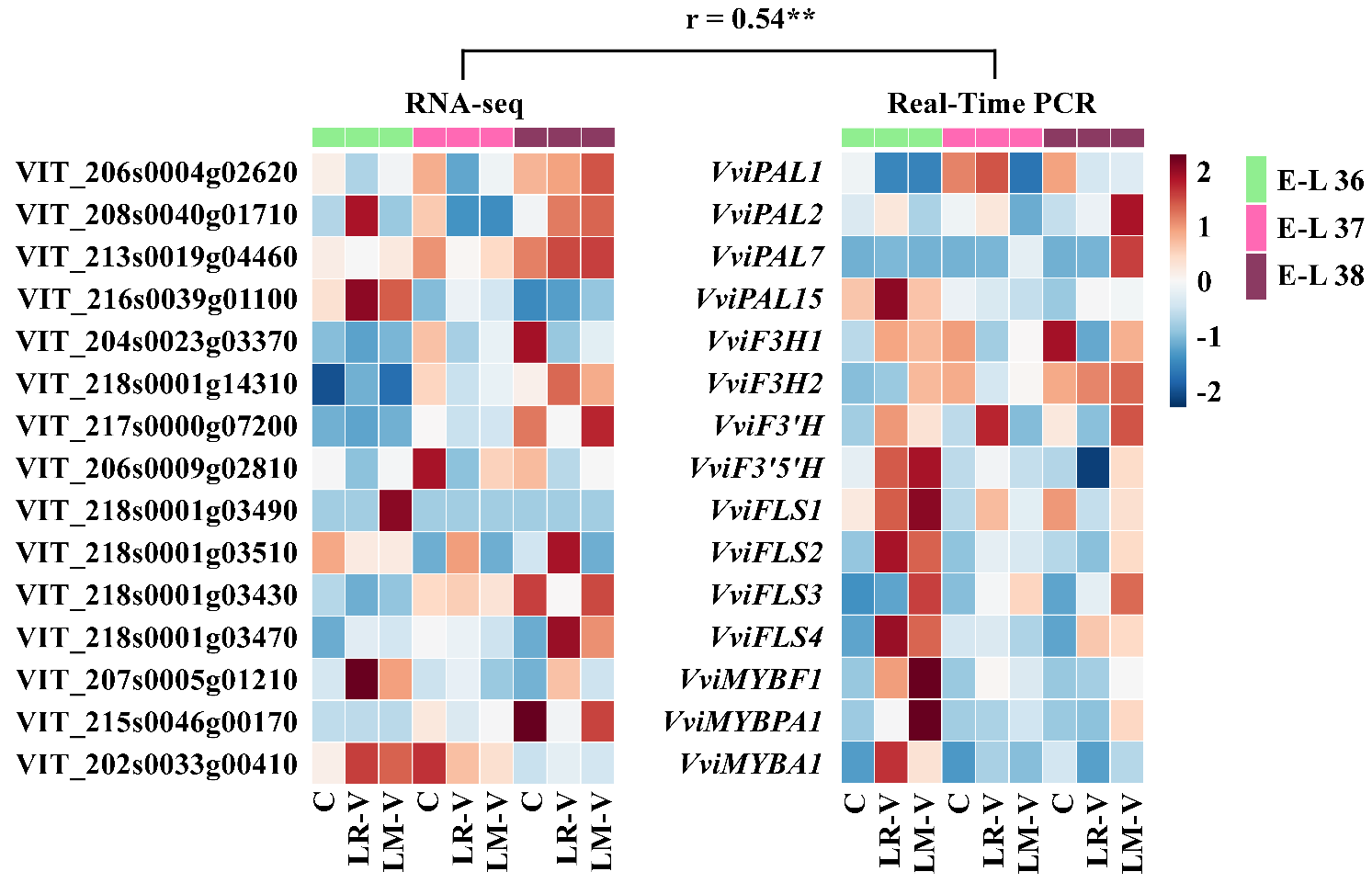


**Figure S2.** Validation of RNA-seq results by quantitative real-time PCR. The left-hand heatmap indicates the average FPKM values of the RNA-seq analysis. The right-hand heatmap indicates the average value of relative expression from three independent real-time PCR experiments. Correlation is significant at the 0.01 level. C: control group; LR-V: leaf removal at véraison; LM-V: leaf moving at véraison.

## Supplementary Tables

**Table S1.** Meteorological data of the study area during the developmental period of grape berries in 2011-2013.

| Developmental  phases | Days | | | Growing degree days (°C) | | | Sunshine duration (h) | | | Temperature (°C) | | | | | | | | | Rainfall (mm) | | | Relative humidity (%) | | |
| --- | --- | --- | --- | --- | --- | --- | --- | --- | --- | --- | --- | --- | --- | --- | --- | --- | --- | --- | --- | --- | --- | --- | --- | --- |
|  |  |  |  |  |  |  |  |  |  | Average | | | Maximum | | | Minimum | | |  |  |  |  |  |  |
|  | 2011 | 2012 | 2013 | 2011 | 2012 | 2013 | 2011 | 2012 | 2013 | 2011 | 2012 | 2013 | 2011 | 2012 | 2013 | 2011 | 2012 | 2013 | 2011 | 2012 | 2013 | 2011 | 2012 | 2013 |
| flowering | 7 | 7 | 7 | 102.9 | 113.5 | 96.7 | 70.3 | 70.8 | 82.4 | 24.7 | 26.2 | 23.8 | 31.1 | 31.2 | 30.9 | 19.7 | 20.9 | 18.5 | 0.1 | 0.1 | 1.1 | 33.9 | 30.7 | 32.1 |
| green | 52 | 47 | 42 | 850.5 | 785.6 | 624.1 | 586.6 | 497.7 | 451.7 | 26.4 | 26.7 | 24.9 | 32.5 | 32.6 | 30.8 | 20.7 | 21.4 | 19.5 | 49.6 | 35.9 | 38.7 | 40.4 | 40.7 | 34.6 |
| véraison | 18 | 21 | 23 | 295.9 | 347.4 | 373.2 | 184.8 | 240.0 | 237.4 | 26.4 | 26.5 | 26.2 | 32.8 | 32.8 | 32.6 | 20.6 | 20.3 | 19.5 | 6.2 | 10.2 | 11.3 | 40.1 | 41.6 | 38.6 |
| ripening | 38 | 39 | 41 | 445.9 | 480.8 | 415.8 | 394.7 | 399.8 | 415.7 | 21.7 | 22.3 | 20.1 | 28.4 | 28.8 | 27.8 | 15.4 | 15.7 | 13.0 | 35.0 | 20.1 | 21.9 | 42.9 | 42.4 | 40.9 |

**Table S2.** Microclimate conditions of fruiting zone in control and cluster sunlight exposure treatments during the 2012 and 2013 growing seasons.

|  | Photosynthetically active radiation (µmol/m^2^·s) | | | | Solar radiation (µmol·m^-2^·s^-1^) | | | | Average temperature (°C) | | | | Relative humidity (%) | | | |
| --- | --- | --- | --- | --- | --- | --- | --- | --- | --- | --- | --- | --- | --- | --- | --- | --- |
|  | C | LR | HLR | LM | C | LR | HLR | LM | C | LR | HLR | LM | C | LR | HLR | LM |
| 2012 |  |  |  |  |  |  |  |  |  |  |  |  |  |  |  |  |
| Stage I | 14.78 | 38.36 |  |  | 167.4 | 334.9 |  |  | 25.36 | 26.31 |  |  | 58.67 | 55.30 |  |  |
| Stage II | 19.02 | 64.45 | 51.94 | 63.91 | 238.9 | 288.9 | 359.0 | 279.8 | 24.64 | 25.08 | 25.01 | 25.06 | 55.78 | 54.42 | 54.23 | 53.63 |
| Stage III | 11.61 | 58.89 | 43.40 | 39.01 | 145.6 | 182.5 | 222.5 | 167.4 | 18.82 | 19.06 | 19.19 | 19.11 | 60.72 | 59.88 | 59.00 | 58.79 |
| 2013 |  |  |  |  |  |  |  |  |  |  |  |  |  |  |  |  |
| Stage I | 11.84 | 52.63 |  |  | 205.7 | 166.5 |  |  | 23.86 | 23.99 |  |  | 62.70 | 60.77 |  |  |
| Stage II | 20.08 | 69.44 | 22.60 | 33.88 | 154.2 | 292.1 | 180.6 | 205.7 | 22.60 | 23.03 | 22.77 | 22.96 | 69.44 | 66.57 | 67.07 | 66.24 |
| Stage III | 10.67 | 54.75 | 22.07 | 35.15 | 141.1 | 247.1 | 147.4 | 190.2 | 16.85 | 17.30 | 17.08 | 17.15 | 64.75 | 61.23 | 61.68 | 61.17 |

Stage I, II and III represent the herbaceous phase, véraison and the maturation phase of grape berries, respectively. C: control group; LR: leaf removal; HLR: half leaf removal; LM: leaf moving.

**Table S3.** List of the primers used for quantitative real-time PCR validation experiments.

| Gene name | Gene ID (CRIBI, V2) | Annotation | Primers (5′ to 3′) | Reference |
| --- | --- | --- | --- | --- |
| *VviPAL1* | VIT_206s0004g02620 | *phenylalanine ammonia-lyase* | Forward: CTCACACCACAACGGCAACG  Revers: CGCCACCATTCTCTTCACCTC | Sun et al. 2016 |
| *VviPAL2* | VIT_208s0040g01710 | *phenylalanine ammonia-lyase* | Forward: CCACCCATCAAGATTCTGCGAG  Revers: CATTGTTCAGTGCGTGTTCTACCA | Sun et al. 2016 |
| *VviPAL7* | VIT_213s0019g04460 | *phenylalanine ammonia-lyase* | Forward: GAAGAACCAAACAAGGCGGAG  Revers: AGAGGCAAGCAAGGGGTAATG | Sun et al. 2016 |
| *VviPAL15* | VIT_216s0039g01100 | *phenylalanine ammonia-lyase* | Forward: CAACTCTGTGAATGATAACCCCTT  Revers: TGCTCCCTTGAAACCATAGTCC | Sun et al. 2016 |
| *VviF3H1* | VIT_204s0023g03370 | *flavanone 3-hydroxylase* | Forward: CCAATCATAGCAGACTGTCC  Revers: TCAGAGGATACACGGTTGCC | Azuma et al. 2012 |
| *VviF3H2* | VIT_218s0001g14310 | *flavanone 3-hydroxylase* | Forward: CTGTGGTGAACTCCGACTGC  Revers: CAAATGTTATGGGCTCCTCC | Azuma et al. 2012 |
| *VviF3'H* | VIT_217s0000g07200 | *flavonoid 3'-hydroxylase* | Forward: ACGGCTACCACATCCCCAAAAA  Revers: CCCCGAATGGAATCACTTCAAAAT | Sun et al. 2015b |
| *VviF3'5'H* | VIT_206s0009g02810 | *flavonoid 3',5'-hydroxylase* | Forward: GAAGTTCGACTGGTTATTAACAAAGAT  Revers: AGGAGGAGTGCTTTAATGTTGGTA | Castellarin et al. 2006 |
| *VviFLS1* | VIT_218s0001g03490 | *flavonol synthase* | Forward: AATCCTCCTTCTTACAGGGA  Revers: AGCCCTAACCCTACCGACAA | Fujita et al. 2006 |
| *VviFLS2* | VIT_218s0001g03510 | *flavonol synthase* | Forward: AACCCACCTTCGTACAGGGC  Revers: CCTAACCCTAATGACAGCAA | Fujita et al. 2006 |
| *VviFLS3* | VIT_218s0001g03430 | *flavonol synthase* | Forward: AACCAAGATGACTAAGAACC  Revers: CTTCTGTGACTTCCCTGTAG | Fujita et al. 2006 |
| *VviFLS4* | VIT_218s0001g03470 | *flavonol synthase* | Forward: AAACCACCTACTTACAGAGC  Revers: ACCTAACCCCAGTGACAGAC | Fujita et al. 2006 |
| *VviMYBF1* | VIT_207s0005g01210 | *transcription factor myb12* | Forward: GGAGGTTGAGGGGTTGTG  Revers: AAGTTGGGGAAGAGCAGGAG | Czemmel et al. 2009 |
| *Vvi**MYBPA1* | VIT_215s0046g00170 | *transcription factor mybpa1* | Forward: AGATCAACTGGTTATGCTTGCT  Revers: AACACAAATGTACATCGCACAC | Bogs et al. 2007 |
| *VviMYBA1* | VIT_202s0033g00410 | *transcription factor myba1* | Forward: GCAAGCCTCAGGACAGAAGAA  Revers: ATCCCAGAAGCCCACATCAA | Shimazaki et al. 2011 |
| *VviUbiquitin1* | VIT_216s0098g01190 | *ubiquitin fusion protein* | Forward: GTGGTATTATTGAGCCATCCTT  Revers: AACCTCCAATCCAGTCATCTAC | Downey et al. 2003 |
| *Vviβ-Actin* | VIT_204s0044g00580 | *actin protein* | Forward: CTTGCATCCCTCAGCACCTT  Revers: TCCTGTGGACAATGGATGGA | Reid et al. 2006 |

**Table S4.** Statistical analysis of the physicochemical indexes and phenolic accumulations in different cluster sunlight exposed grape berries during development over three seasons.

| Parameter | Year | Developmental stage | C | LR-PS | LR-V | LR-AV | HLR-V | HLR-AV | LM-V | LM-AV |
| --- | --- | --- | --- | --- | --- | --- | --- | --- | --- | --- |
| Berry fresh weight (g) | 2011 | E-L 31 | 0.60 ± 0.06a | 0.60 ± 0.05a |  |  |  |  |  |  |
|  |  | E-L 33 | 0.64 ± 0.04a | 0.67 ± 0.04a |  |  |  |  |  |  |
|  |  | E-L 35 | 0.81 ± 0.05a | 0.85 ± 0.01a |  |  |  |  |  |  |
|  |  | E-L 36 | 0.85 ± 0.06a | 1.02 ± 0.08b | 0.93 ± 0.02a |  | 1.05 ± 0.09b |  | 1.05 ± 0.04b |  |
|  |  | E-L 37 | 1.08 ± 0.08a | 1.23 ± 0.05c | 1.13 ± 0.05ab |  | 1.18 ± 0.09bc |  | 1.10 ± 0.02ab |  |
|  |  | E-L 38 | 1.25 ± 0.02b | 1.10 ± 0.06a | 1.33 ± 0.09bc | 1.14 ± 0.06a | 1.33 ± 0.10bc | 1.14 ± 0.06a | 1.30 ± 0.01bc | 1.36 ± 0.14c |
|  | 2012 | E-L 29 | 0.59 ± 0.02 |  |  |  |  |  |  |  |
|  |  | E-L 31 | 0.61 ± 0.01b | 0.46 ± 0.07a |  |  |  |  |  |  |
|  |  | E-L 33 | 0.76 ± 0.05a | 0.73 ± 0.01a |  |  |  |  |  |  |
|  |  | E-L 35 | 0.78 ± 0.02a | 0.94 ± 0.03b |  |  |  |  |  |  |
|  |  | E-L 36 | 1.04 ± 0.02ab | 1.06 ± 0.02c | 1.03 ± 0.04ab |  | 0.96 ± 0.05a |  | 1.16 ± 0.07d |  |
|  |  | E-L 37 | 1.03 ± 0.11a | 1.16 ± 0.03b | 1.16 ± 0.02b |  | 1.04 ± 0.03a |  | 1.29 ± 0.05c |  |
|  |  | E-L 38 | 1.01 ± 0.04b | 1.22 ± 0.03e | 0.99 ± 0.03a | 1.11 ± 0.06 | 1.05 ± 0.01bc | 1.13 ± 0.03cd | 1.18 ± 0.05de | 1.23 ± 0.02e |
|  | 2013 | E-L 29 | 0.50 ± 0.01 |  |  |  |  |  |  |  |
|  |  | E-L 31 | 0.64 ± 0.01a | 0.65 ± 0.11a |  |  |  |  |  |  |
|  |  | E-L 33 | 0.62 ± 0.03a | 1.03 ± 0.05b |  |  |  |  |  |  |
|  |  | E-L 35 | 0.76 ± 0.02a | 0.75 ± 0.04a |  |  |  |  |  |  |
|  |  | E-L 36 | 0.86 ± 0.08a | 0.88 ± 0.07a | 0.90 ± 0.04a |  | 0.97 ± 0.03a |  | 0.88 ± 0.05a |  |
|  |  | E-L 37 | 1.06 ± 0.07bc | 0.94 ± 0.06a | 1.05 ± 0.08abc |  | 1.11 ± 0.05c |  | 0.99 ± 0.03ab |  |
|  |  | E-L 38 | 1.09 ± 0.10a | 1.00 ± 0.08a | 1.06 ± 0.07a | 1.14 ± 0.11a | 1.10 ± 0.05a | 1.11 ± 0.09a | 1.06 ± 0.10a | 1.11 ± 0.06a |
| Total soluble solids (^o^Brix) | 2011 | E-L 31 | 4.10 ± 0.01 a | 4.10 ± 0.01 a |  |  |  |  |  |  |
|  |  | E-L 33 | 4.60 ± 0.01a | 4.8 ± 0.10b |  |  |  |  |  |  |
|  |  | E-L 35 | 6.97 ± 0.06b | 5.13 ± 0.06a |  |  |  |  |  |  |
|  |  | E-L 36 | 11.40 ± 0.01b | 12.70 ± 0.10e | 12.13 ± 0.12d |  | 11.80 ± 0.01c |  | 10.63 ± 0.12a |  |
|  |  | E-L 37 | 17.00 ± 0.01b | 16.70 ± 0.01a | 18.23 ± 0.15c |  | 19.50 ± 0.01d |  | 19.50 ± 0.01d |  |
|  |  | E-L 38 | 22.93 ± 0.06b | 26.83 ± 0.06 | 23.33 ± 0.06c | 24.17 ± 0.29d | 22.40 ± 0.01a | 24.10 ± 0.01d | 24.07 ± 0.12d | 24.80 ± 0.10e |
|  | 2012 | E-L 29 | 3.3 ± 0.26 |  |  |  |  |  |  |  |
|  |  | E-L 31 | 4.07 ± 0.21a | 4.6 ± 0.01b |  |  |  |  |  |  |
|  |  | E-L 33 | 4.43 ± 0.06a | 4.83 ± 0.06b |  |  |  |  |  |  |
|  |  | E-L 35 | 6.77 ± 0.06a | 10.33 ± 0.06b |  |  |  |  |  |  |
|  |  | E-L 36 | 16.87 ± 0.06a | 16.87 ± 0.06a | 17.07 ± 0.06a |  | 17.77 ± 0.06a |  | 18.73 ± 0.10b |  |
|  |  | E-L 37 | 20.03 ± 0.06b | 20.67 ± 0.06c | 19.37 ± 0.06a |  | 21.17 ± 0.06d |  | 20.13 ± 0.06b |  |
|  |  | E-L 38 | 26.20 ± 0.61c | 24.50 ± 0.10a | 25.80 ± 0.10bc | 25.50 ± 0.61b | 25.47 ± 0.06b | 25.67 ± 0.06bc | 26.00 ± 0.26bc | 24.33 ± 0.06a |
|  | 2013 | E-L 29 | 4.67 ± 0.06 |  |  |  |  |  |  |  |
|  |  | E-L 31 | 3.90 ± 0.17a | 3.97 ± 0.21a |  |  |  |  |  |  |
|  |  | E-L 33 | 4.03 ± 0.31a | 4.17 ± 0.21a |  |  |  |  |  |  |
|  |  | E-L 35 | 6.53 ± 1.45a | 8.27 ± 2.25a |  |  |  |  |  |  |
|  |  | E-L 36 | 11.37 ± 0.38a | 11.63 ± 0.67a | 11.50 ± 1.47a |  | 12.17 ± 0.40a |  | 11.73 ± 0.93a |  |
|  |  | E-L 37 | 13.70 ± 0.87a | 14.43 ± 0.23a | 14.00 ± 0.87a |  | 13.53 ± 0.12a |  | 14.47 ± 0.32a |  |
|  |  | E-L 38 | 22.33 ± 1.30a | 22.43 ± 0.85a | 21.70 ± 0.79a | 22.20 ± 0.56a | 22.07 ± 1.19a | 22.47 ± 0.86a | 21.60 ± 0.87a | 22.77 ± 0.50a |
| Titratable acidity (g/L) | 2011 | E-L 31 | 31.30 ± 0.37a | 31.30 ± 0.37a |  |  |  |  |  |  |
|  |  | E-L 33 | 35.00 ± 0.18a | 37.79 ± 0.21b |  |  |  |  |  |  |
|  |  | E-L 35 | 30.67 ± 0.42a | 34.78 ± 0.36b |  |  |  |  |  |  |
|  |  | E-L 36 | 20.77 ± 0.42d | 16.42 ± 0.84c | 14.97 ± 0.42b |  | 13.04 ± 0.01a |  | 21.61 ± 0.21d |  |
|  |  | E-L 37 | 7.97 ± 0.01c | 5.80 ± 0.01a | 7.73 ± 0.21b |  | 14.59 ± 0.01d |  | 7.97 ± 0.01c |  |
|  |  | E-L 38 | 3.65 ± 0.04b | 3.86 ± 0.21b | 3.62 ± 0.01b | 4.47 ± 0.21c | 4.83 ± 0.21d | 3.26 ± 0.01a | 4.83 ± 0.21d | 3.62 ± 0.01b |
|  | 2012 | E-L 29 | 32.81 ± 0.07 |  |  |  |  |  |  |  |
|  |  | E-L 31 | 31.85 ± 0.60a | 32.91 ± 0.01a |  |  |  |  |  |  |
|  |  | E-L 33 | 31.85 ± 0.60a | 32.18 ± 0.06a |  |  |  |  |  |  |
|  |  | E-L 35 | 29.89 ± 0.06a | 19.58 ± 0.06a |  |  |  |  |  |  |
|  |  | E-L 36 | 15.58 ± 0.20e | 13.77 ± 0.01c | 13.31 ± 0.02b |  | 11.68 ± 0.31a |  | 14.21 ± 0.02d |  |
|  |  | E-L 37 | 10.27 ± 0.01d | 9.21 ± 0.06c | 6.86 ± 0.06b |  | 8.94 ± 0.03c |  | 5.05 ± 0.56a |  |
|  |  | E-L 38 | 4.73 ± 0.19a | 5.07 ± 0.50a | 4.27 ± 0.15a | 4.48 ± 0.14a | 4.69 ± 0.41a | 4.18 ± 0.98a | 4.46 ± 0.09a | 4.26 ± 0.64a |
|  | 2013 | E-L 29 | 38.72 ± 1.28 |  |  |  |  |  |  |  |
|  |  | E-L 31 | 31.04 ± 1.28a | 31.64 ± 1.82a |  |  |  |  |  |  |
|  |  | E-L 33 | 32.13 ± 1.24a | 31.40 ± 2.33a |  |  |  |  |  |  |
|  |  | E-L 35 | 30.03 ± 1.58a | 28.71 ± 2.56a |  |  |  |  |  |  |
|  |  | E-L 36 | 16.79 ± 1.46a | 17.10 ± 1.76a | 16.68 ± 2.34a |  | 16.15 ± 1.73a |  | 17.45 ± 1.17a |  |
|  |  | E-L 37 | 12.02 ± 0.56a | 10.89 ± 1.64a | 12.30 ± 11.44a |  | 11.76 ± 0.59a |  | 11.48 ± 1.05a |  |
|  |  | E-L 38 | 4.71 ± 0.51ab | 4.34 ± 0.40a | 4.83 ± 0.55ab | 4.47 ± 0.12ab | 5.15 ± 0.41b | 4.81 ± 0.47ab | 4.79 ± 0.43ab | 4.71 ± 0.16ab |
| Hydroxycinnamic acids (mg/kg FW) | 2011 | E-L 31 | 132.35 ± 2.78b | 88.91 ± 2.76a |  |  |  |  |  |  |
|  |  | E-L 33 | 73.97 ± 0.94b | 67.14 ± 2.87a |  |  |  |  |  |  |
|  |  | E-L 35 | 47.85 ± 0.49b | 30.30 ± 1.31a |  |  |  |  |  |  |
|  |  | E-L 36 | 41.97 ± 1.43d | 41.26 ± 1.57d | 36.78 ± 0.88c |  | 27.67 ± 0.63b |  | 22.99 ± 0.03a |  |
|  |  | E-L 37 | 23.97 ± 0.74a | 30.56 ± 0.43c | 35.83 ± 0.65d |  | 98.07 ± 2.22e |  | 27.03 ± 0.63b |  |
|  |  | E-L 38 | 45.63 ± 0.95c | 33.34 ± 3.13a | 48.05 ± 0.90cd | 38.77 ± 1.32b | 49.85 ± 1.17d | 50.20 ± 1.56d | 60.48 ± 3.27e | 91.49 ± 2.46f |
|  | 2012 | E-L 29 | 194.08 ± 12.78 |  |  |  |  |  |  |  |
|  |  | E-L 31 | 95.12 ± 11.33b | 54.11 ± 9.66a |  |  |  |  |  |  |
|  |  | E-L 33 | 189.81 ± 17.52b | 46.47 ± 10.16a |  |  |  |  |  |  |
|  |  | E-L 35 | 103.29 ± 15.52b | 48.64 ± 4.53a |  |  |  |  |  |  |
|  |  | E-L 36 | 38.11 ± 1.71a | 55.16 ± 1.28b | 108.23 ± 4.41d |  | 73.17 ± 2.23c |  | 130.99 ± 3.94e |  |
|  |  | E-L 37 | 58.71 ± 1.32bc | 62.92 ± 8.95c | 44.06 ± 2.18a |  | 51.00 ± 1.61ab |  | 126.46 ± 4.32d |  |
|  |  | E-L 38 | 63.13 ± 5.24a | 58.06 ± 8.32a | 106.32 ± 1.38cd | 49.40 ± 4.59a | 84.72 ± 2.16ab | 121.95 ± 4.67d | 131.64 ± 16.70d | 77.58 ± 15.52ab |
|  | 2013 | E-L 29 | 111.60 ± 10.39 |  |  |  |  |  |  |  |
|  |  | E-L 31 | 119.14 ± 19.70a | 119.74 ± 3.75a |  |  |  |  |  |  |
|  |  | E-L 33 | 51.52 ± 0.71a | 67.63 ± 4.87b |  |  |  |  |  |  |
|  |  | E-L 35 | 33.37 ± 5.22a | 97.45 ± 2.03b |  |  |  |  |  |  |
|  |  | E-L 36 | 46.24 ± 3.31c | 26.50 ± 0.34a | 58.81 ± 0.09d |  | 41.93 ± 0.66b |  | 55.99 ± 1.78d |  |
|  |  | E-L 37 | 56.22 ± 4.48a | 109.02 ± 1.68b | 69.19 ± 8.94a |  | 104.45 ± 2.50b |  | 52.86 ± 1.94a |  |
|  |  | E-L 38 | 36.39 ± 0.84a | 57.43 ± 0.62c | 88.22 ± 1.85f | 66.83 ± 1.30d | 70.02 ± 0.79e | 87.88 ± 1.34g | 89.18 ± 2.48g | 42.74 ± 1.43b |
| Favonoids (mg/kg FW) | 2011 | E-L 31 | 1381.73 ± 51.18a | 1659.90 ± 80.80b |  |  |  |  |  |  |
|  |  | E-L 33 | 1361.38 ± 25.91a | 1436.97 ± 16.15b |  |  |  |  |  |  |
|  |  | E-L 35 | 1107.35 ± 24.73b | 934.53 ± 29.29a |  |  |  |  |  |  |
|  |  | E-L 36 | 1207.15 ± 15.99c | 938.40 ± 6.83b | 1438.17 ± 31.30d |  | 793.95 ± 15.59a |  | 779.16 ± 22.58a |  |
|  |  | E-L 37 | 1214.06 ± 52.69bc | 1121.49 ± 11.07a | 1132.41 ± 64.64ab |  | 1284.82 ± 63.99c |  | 1418.46 ± 13.77d |  |
|  |  | E-L 38 | 1496.11 ± 26.96cd | 1253.99 ± 79.20a | 1348.80 ± 54.87ab | 1416.83 ± 68.00bc | 1295.01 ± 62.28ab | 1584.96 ± 17.56d | 1726.12 ± 129.50e | 1480.18 ± 74.18cd |
|  | 2012 | E-L 29 | 1875 ± 48.14 |  |  |  |  |  |  |  |
|  |  | E-L 31 | 1805.98 ± 45.73a | 2401.47 ± 76.62b |  |  |  |  |  |  |
|  |  | E-L 33 | 1224.55 ± 11.11b | 929.63 ± 25.31a |  |  |  |  |  |  |
|  |  | E-L 35 | 1259.00 ± 10.02a | 1752.43 ± 6.47b |  |  |  |  |  |  |
|  |  | E-L 36 | 1608.80 ± 33.34b | 1723.10 ± 93.05b | 2030.69 ± 22.37c |  | 1404.13 ± 97.28a |  | 1928.16 ± 31.11c |  |
|  |  | E-L 37 | 1718.91 ± 36.52ab | 1661.14 ± 9.17a | 1889.94 ± 89.95c |  | 1618.56 ± 88.69a |  | 1809.89 ± 84.74bc |  |
|  |  | E-L 38 | 2010.90 ± 66.51e | 1878.68 ± 98.63bcd | 2031.83 ± 114.21de | 1792.70 ± 112.67bc | 1702.23 ± 83.66b | 1897.41 ± 108.45cd | 1805.37 ± 72.01bc | 1243.01 ± 114.56a |
|  | 2013 | E-L 29 | 2635.94 ± 297.12 |  |  |  |  |  |  |  |
|  |  | E-L 31 | 2379.61 ± 127.04a | 2391.99 ± 156.07a |  |  |  |  |  |  |
|  |  | E-L 33 | 2421.74 ± 300.32a | 2274.32 ± 491.07a |  |  |  |  |  |  |
|  |  | E-L 35 | 2394.36 ± 294.49a | 2251.93 ± 238.61a |  |  |  |  |  |  |
|  |  | E-L 36 | 2014.81 ± 15.66ab | 2478.59 ± 143.94c | 2079.59 ± 77.70b |  | 1696.95 ± 389.13a |  | 2028.05 ± 91.52ab |  |
|  |  | E-L 37 | 1888.62 ± 65.45a | 1941.08 ± 426.45a | 1921.35 ± 42.73a |  | 2158.82 ± 315.72a |  | 1969.64 ± 102.22a |  |
|  |  | E-L 38 | 2481.49 ± 295.47a | 2500.40 ± 204.37a | 2927.32 ± 178.16b | 2845.28 ± 221.94ab | 2703.54 ± 153.49ab | 2590.58 ± 153.59ab | 2571.98 ± 66.11ab | 2543.41 ± 216.12a |
| Flavan-3-ols (mg/kg FW) | 2011 | E-L 31 | 1368.54 ± 51.19a | 1680.04 ± 80.95b |  |  |  |  |  |  |
|  |  | E-L 33 | 1347.63 ± 27.07a | 1397.49 ± 16.01a |  |  |  |  |  |  |
|  |  | E-L 35 | 1039.64 ± 26.03b | 829.84 ± 22.28a |  |  |  |  |  |  |
|  |  | E-L 36 | 1042.86 ± 14.23c | 771.36 ± 11.33b | 1298.43 ± 23.21d |  | 660.72 ± 12.78a |  | 657.06 ± 25.66a |  |
|  |  | E-L 37 | 721.95 ± 42.17c | 525.40 ± 5.48a | 540.70 ± 11.67a |  | 701.09 ± 73.55bc |  | 636.91 ± 12.02b |  |
|  |  | E-L 38 | 760.02 ± 17.27d | 421.99 ± 14.45a | 437.14 ± 11.31ab | 431.79 ± 3.63ab | 475.51 ± 15.34b | 672.26 ± 33.11c | 726.49 ± 40.82d | 740.51 ± 45.95d |
|  | 2012 | E-L 29 | 1851.14 ± 45.66 |  |  |  |  |  |  |  |
|  |  | E-L 31 | 1720.51 ± 45.68a | 2361.43 ± 77.06b |  |  |  |  |  |  |
|  |  | E-L 33 | 1178.95 ± 10.64b | 897.65 ± 24.99a |  |  |  |  |  |  |
|  |  | E-L 35 | 1102.51 ± 14.45a | 1572.26 ± 25.36b |  |  |  |  |  |  |
|  |  | E-L 36 | 1108.36 ± 16.13b | 1082.87 ± 43.25b | 1304.25 ± 30.94c |  | 924.91 ± 84.51a |  | 1222.83 ± 23.30c |  |
|  |  | E-L 37 | 958.97 ± 16.38a | 992.41 ± 6.75ab | 1117.38 ± 114.19b |  | 1038.09 ± 94.64ab |  | 1016.06 ± 25.51ab |  |
|  |  | E-L 38 | 1034.33 ± 45.42c | 937.88 ± 48.01bc | 999.97 ± 50.25c | 968.58 ± 31.50bc | 877.87 ± 49.06b | 994.45 ± 108.54c | 883.60 ± 7.21b | 703.04 ± 66.02a |
|  | 2013 | E-L 29 | 2632.6 ± 297.31 |  |  |  |  |  |  |  |
|  |  | E-L 31 | 2372.62 ± 1225.53a | 2391.99 ± 156.07a |  |  |  |  |  |  |
|  |  | E-L 33 | 2396.66 ± 300.64a | 2255.37 ± 491.35a |  |  |  |  |  |  |
|  |  | E-L 35 | 2337.07 ± 302.36a | 2020.65 ± 251.26a |  |  |  |  |  |  |
|  |  | E-L 36 | 1909.43 ± 41.00c | 2187.44 ± 170.53c | 1782.71 ± 110.92ab |  | 1736.51 ± 274.40a |  | 1848.99 ± 96.77ab |  |
|  |  | E-L 37 | 1489.82 ± 67.43a | 1396.73 ± 405.92a | 1442.58 ± 41.71a |  | 1602.58 ± 48.28a |  | 1577.97 ± 99.01a |  |
|  |  | E-L 38 | 1395.20 ± 179.61ab | 1146.42 ± 13.78a | 1716.54 ± 174.32c | 1583.73 ± 151.60bc | 1694.50 ± 95.71c | 1576.99 ± 201.11bc | 1545.04 ± 114.64bc | 1460.95 ± 187.77bc |
| Anthocyanins (mg/kg FW) | 2011 | E-L 31 | nd | nd |  |  |  |  |  |  |
|  |  | E-L 33 | nd | nd |  |  |  |  |  |  |
|  |  | E-L 35 | 138.41 ± 3.42b | 121.17 ± 6.40a |  |  |  |  |  |  |
|  |  | E-L 36 | 445.06 ± 11.99a | 544.70 ± 14.60b | 540.04 ± 57.98b |  | 549.72 ± 29.09b |  | 715.66 ± 5.44c |  |
|  |  | E-L 37 | 615.68 ± 84.66ab | 665.74 ± 67.04bc | 520.25 ± 59.89a |  | 772.28 ± 10.39c |  | 683.02 ± 23.76bc |  |
|  |  | E-L 38 | 639.17 ± 42.29a | 716.55 ± 65.95ab | 801.53 ± 42.96bc | 832.38 ± 69.56bc | 708.69 ± 88.15ab | 812.93 ± 24.93bc | 874.04 ± 93.46c | 666.60 ± 101.12a |
|  | 2012 | E-L 29 | nd |  |  |  |  |  |  |  |
|  |  | E-L 31 | nd | nd |  |  |  |  |  |  |
|  |  | E-L 33 | nd | nd |  |  |  |  |  |  |
|  |  | E-L 35 | 107.45 ± 4.71a | 107.17 ± 20.35a |  |  |  |  |  |  |
|  |  | E-L 36 | 442.19 ± 18.21ab | 499.61 ± 37.04b | 602.02 ± 37.47c |  | 388.59 ± 30.27a |  | 613.69 ± 59.39c |  |
|  |  | E-L 37 | 673.00 ± 16.87b | 530.95 ± 16.53a | 622.28 ± 43.96b |  | 488.83 ± 6.54a |  | 651.52 ± 63.96b |  |
|  |  | E-L 38 | 965.64 ± 40.02d | 748.55 ± 60.61bc | 864.99 ± 90.65cd | 700.79 ± 108.53b | 726.08 ± 53.14b | 754.94 ± 11.49bc | 785.80 ± 70.38bc | 457.48 ± 42.72a |
|  | 2013 | E-L 29 | nd |  |  |  |  |  |  |  |
|  |  | E-L 31 | nd | nd |  |  |  |  |  |  |
|  |  | E-L 33 | nd | nd |  |  |  |  |  |  |
|  |  | E-L 35 | 4.70 ± 0.04a | 73.06 ± 10.89b |  |  |  |  |  |  |
|  |  | E-L 36 | 114.72 ± 34.52a | 219.65 ± 30.99bc | 273.09 ± 72.42c |  | 196.64 ± 20.92bc |  | 169.21 ± 5.55ab |  |
|  |  | E-L 37 | 356.92 ± 5.90a | 451.10 ± 49.25b | 418.90 ± 28.53ab |  | 371.14 ± 57.96a |  | 365.95 ± 7.19a |  |
|  |  | E-L 38 | 1008.37 ± 176.26abc | 1195.77 ± 188.76c | 1052.56 ± 9.62abc | 1134.47 ± 79.54bc | 931.25 ± 54.74ab | 914.46 ± 91.23a | 932.38 ± 70.75ab | 983.71 ± 18.29ab |
| Flavonols (mg/kg FW) | 2011 | E-L 31 | 13.19 ± 0.02a | 15.86 ± 0.74b |  |  |  |  |  |  |
|  |  | E-L 33 | 13.75 ± 1.17a | 39.48 ± 0.15b |  |  |  |  |  |  |
|  |  | E-L 35 | 15.14 ± 0.26a | 33.24 ± 0.86b |  |  |  |  |  |  |
|  |  | E-L 36 | 25.88 ± 0.06c | 45.86 ± 0.42d | 23.27 ± 0.33b |  | 20.61 ± 2.85a |  | 19.44 ± 0.25a |  |
|  |  | E-L 37 | 47.05 ± 7.64b | 51.39 ± 1.95b | 51.67 ± 0.07b |  | 34.01 ± 3.96a |  | 65.90 ± 5.36c |  |
|  |  | E-L 38 | 96.92 ± 1.46b | 115.45 ± 2.81cd | 110.12 ± 5.78bcd | 152.66 ± 4.39e | 110.81 ± 13.87bcd | 99.76 ± 10.01bc | 125.59 ± 5.91d | 73.08 ± 18.49a |
|  | 2012 | E-L 29 | 24.03 ± 2.66 |  |  |  |  |  |  |  |
|  |  | E-L 31 | 85.46 ± 0.28b | 40.04 ± 0.48a |  |  |  |  |  |  |
|  |  | E-L 33 | 45.60 ± 0.56b | 31.98 ± 1.77a |  |  |  |  |  |  |
|  |  | E-L 35 | 49.05 ± 0.62a | 73.00 ± 1.24b |  |  |  |  |  |  |
|  |  | E-L 36 | 58.25 ± 4.85a | 140.61 ± 15.13d | 124.42 ± 4.15c |  | 90.63 ± 5.35b |  | 91.64 ± 8.74b |  |
|  |  | E-L 37 | 86.95 ± 11.27a | 137.77 ± 7.00b | 150.28 ± 12.91b |  | 91.63 ± 2.77a |  | 142.31 ± 7.92b |  |
|  |  | E-L 38 | 101.93 ± 4.50b | 192.25 ± 9.08f | 166.88 ± 6.19e | 123.32 ± 5.60c | 98.27 ± 4.38b | 148.02 ± 9.53d | 125.97 ± 1.50c | 82.49 ± 7.22a |
|  | 2013 | E-L 29 | 3.34 ± 0.64 |  |  |  |  |  |  |  |
|  |  | E-L 31 | 6.99 ± 2.15a | 48.18 ± 0.23b |  |  |  |  |  |  |
|  |  | E-L 33 | 25.08 ± 0.37a | 117.29 ± 13.14b |  |  |  |  |  |  |
|  |  | E-L 35 | 52.60 ± 8.00a | 174.97 ± 15.33b |  |  |  |  |  |  |
|  |  | E-L 36 | 17.66 ± 2.57ab | 142.66 ± 24.54c | 23.80 ± 7.08b |  | 18.26 ± 5.25ab |  | 9.86 ± 5.06a |  |
|  |  | E-L 37 | 41.89 ± 19.10ab | 128.49 ± 3.12c | 59.86 ± 7.36b |  | 51.17 ± 9.35b |  | 25.71 ± 8.82a |  |
|  |  | E-L 38 | 77.92 ± 12.17a | 112.00 ± 27.16c | 158.22 ± 18.95c | 127.08 ± 16.68b | 77.79 ± 5.62a | 99.12 ± 2.23a | 94.57 ± 6.37a | 98.74 ± 11.07a |
| Ratio of dhydroxylated to trihydroxylated flavonoids | 2011 | E-L 31 | 1.10 ± 0.02b | 0.68 ± 0.01a |  |  |  |  |  |  |
|  |  | E-L 33 | 1.12 ± 0.04b | 0.82 ± 0.02a |  |  |  |  |  |  |
|  |  | E-L 35 | 0.76 ± 0.02a | 0.88 ± 0.04b |  |  |  |  |  |  |
|  |  | E-L 36 | 0.92 ± 0.03d | 0.83 ± 0.01c | 0.89 ± 0.01d |  | 0.70 ± 0.02a |  | 0.76 ± 0.02b |  |
|  |  | E-L 37 | 0.61 ± 0.01c | 0.39 ± 0.01a | 0.42 ± 0.03b |  | 0.44 ± 0.02b |  | 0.36 ± 0.01a |  |
|  |  | E-L 38 | 0.41 ± 0.02c | 0.25 ± 0.01a | 0.27 ± 0.01a | 0.25 ± 0.01a | 0.27 ± 0.03a | 0.27 ± 0.01a | 0.37 ± 0.01b | 0.39 ± 0.03bc |
|  | 2012 | E-L 29 | 0.65 ± 0.01 |  |  |  |  |  |  |  |
|  |  | E-L 31 | 0.60 ± 0.05b | 0.54 ± 0.03a |  |  |  |  |  |  |
|  |  | E-L 33 | 0.75 ± 0.02a | 0.85 ± 0.04b |  |  |  |  |  |  |
|  |  | E-L 35 | 0.51 ± 0.03a | 0.66 ± 0.04b |  |  |  |  |  |  |
|  |  | E-L 36 | 0.44 ± 0.02ab | 0.48 ± 0.02c | 0.41 ± 0.01a |  | 0.48 ± 0.02bc |  | 0.46 ± 0.02bc |  |
|  |  | E-L 37 | 0.37 ± 0.01a | 0.45 ± 0.01b | 0.45 ± 0.02b |  | 0.43 ± 0.02b |  | 0.43 ± 0.02b |  |
|  |  | E-L 38 | 0.31 ± 0.02a | 0.38 ± 0.02c | 0.38 ± 0.02c | 0.37 ± 0.01c | 0.33 ± 0.02ab | 0.36 ± 0.01bc | 0.38 ± 0.02c | 0.36 ± 0.01bc |
|  | 2013 | E-L 29 | 0.77 ± 0.07 |  |  |  |  |  |  |  |
|  |  | E-L 31 | 0.72 ± 0.01a | 0.77 ± 0.03a |  |  |  |  |  |  |
|  |  | E-L 33 | 0.78 ± 0.04a | 0.71 ± 0.06a |  |  |  |  |  |  |
|  |  | E-L 35 | 0.88 ± 0.07a | 0.76 ± 0.03a |  |  |  |  |  |  |
|  |  | E-L 36 | 0.59 ± 0.02a | 1.02 ± 0.07c | 0.62 ± 0.08ab |  | 0.70 ± 0.04b |  | 0.67 ± 0.06ab |  |
|  |  | E-L 37 | 0.57 ± 0.08a | 0.68 ± 0.03b | 0.73 ± 0.06b |  | 0.57 ± 0.04a |  | 0.54 ± 0.04a |  |
|  |  | E-L 38 | 0.26 ± 0.02abc | 0.25 ± 0.03ab | 0.46 ± 0.04e | 0.34 ± 0.03d | 0.25 ± 0.02a | 0.25 ± 0.01abc | 0.29 ± 0.02ab | 0.29 ± 0.01cd |

Different letters represent significant difference at 0.05 level among treatments in each sampling time point. nd: not detected. C: control group; LR-PS: leaf removal at berry pepper-corn size; LR-V: leaf removal at véraison; LR-AV: leaf removal after véraison; HLR-V: half leaf removal at véraison; HLR-AV: half leaf removal after véraison; LM-V: leaf moving at véraison; LM-AV: leaf moving after véraison leaf removal.

**Table S5.** Correlation analysis between the accumulation of phenolic compounds and the expression of phenolic biosynthesis related genes.

| Metabolites | Gene ID | Enzyme | R-value | P-value |
| --- | --- | --- | --- | --- |
| Total flavonoids | VIT_216s0039g01130 | PAL | 0.86 | 0.01 |
| Total flavonoids | VIT_216s0039g01100 | PAL | 0.84 | 0.01 |
| Total flavonoids | VIT_216s0039g01110 | PAL | 0.84 | 0.01 |
| Total flavonoids | VIT_216s0039g01120 | PAL | 0.8 | 0.01 |
| Total flavonoids | VIT_213s0047g00210 | F3H | 0.77 | 0.05 |
| Total flavonoids | VIT_206s0061g00450 | 4CL | 0.74 | 0.05 |
| Flavonols | VIT_208s0007g00750 | FLS | 0.74 | 0.05 |
| Flavonols | VIT_218s0001g03470 | FLS | 0.73 | 0.05 |

PAL: phenylalanine ammonia-lyase; F3H: flavanone 3-hydroxylase; 4CL: 4-coumarate: CoA ligase; FLS: flavonol synthase.
